# Radiotherapy in Squamous Cell Carcinoma of the Lip: A Systematic Review of Reported Practices, Clinical Outcomes, and Gaps in Consensus

**DOI:** 10.3390/jcm15187090

**Published:** 2026-09-12

**Authors:** Oana Ciobănescu, Anda Elena Crișan, Răzvan Marian Bălan, Virgil Pătrașcu, Andrada-Nemesis Crișan, Cristiana-Iulia Dumitrescu, Mădălin-Cristian Moraru, Suzana Măceș, Mihai Popescu, Daniela Dumitrescu

**Affiliations:** 1 Doctoral School, University of Medicine and Pharmacy of Craiova, 200349 Craiova, Romania; oana.ciobanescu@yahoo.com (O.C.); balan.razvan796@gmail.com (R.M.B.); vm.patrascu@gmail.com (V.P.); mmc9292@gmail.com (M.-C.M.); 2 Department of Radiotherapy, University Emergency County Clinical Hospital Craiova, 200642 Craiova, Romania; 3 Department of Oncology and Radiotherapy, University of Medicine and Pharmacy of Craiova, 200349 Craiova, Romania; 4 SC SPAD Imaging International SRL Center, 200352 Craiova, Romania; macessuzana@yahoo.com (S.M.); mihai_popescu_rad@yahoo.com (M.P.); daniela.dumitrescu@gmail.com (D.D.); 5 CM Galaxy Med SRL Craiova, 200377 Craiova, Romania; an.crisan@yahoo.com; 6 Department of Pharmacology, Faculty of Medicine, University of Medicine and Pharmacy of Craiova, 200349 Craiova, Romania; 7 Department of Radiology and Medical Imaging, University of Medicine and Pharmacy of Craiova, 200349 Craiova, Romania

**Keywords:** squamous cell carcinoma of the lip, radiotherapy, brachytherapy, head and neck cancer, radiation-induced toxicity, functional and aesthetic outcomes, systematic review, ROBINS-I, practice variability, organ preservation

## Abstract

**Background/Objectives:** Squamous cell carcinoma (SCC) of the lip occupies an ambiguous position between cutaneous and oral-cavity oncology and lacks dedicated radiotherapy guidelines, resulting in considerable practice variability. We systematically reviewed the reported radiotherapy practices, clinical outcomes and gaps in consensus at this site. **Methods:** Following PRISMA 2020 and prospectively registered in PROSPERO (CRD420261349994), we searched PubMed/MEDLINE, Scopus, the Cochrane Library (Trials) and ClinicalTrials.gov (20 March 2026). Studies of at least ten patients with lip SCC treated with external beam radiotherapy or brachytherapy were eligible. Risk of bias was assessed with ROBINS-I; the synthesis was narrative, following the Synthesis Without Meta-analysis (SWiM) reporting framework. **Results:** Nine studies (approximately 3904 patients; 1964–2021; six countries) met the criteria. Interstitial brachytherapy was the dominant modality (seven studies), with high local control (approximately 93–97% in modern actuarial series), favourable toxicity and good functional/aesthetic outcomes; external beam radiotherapy served mainly as adjuvant treatment. Doses, fractionation, nodal management and outcome reporting were markedly heterogeneous, and eight of nine studies were at serious risk of bias. **Conclusions:** Radiotherapy—in the identified literature, in effect, largely historical interstitial brachytherapy—appears to be an effective, organ-preserving option in early-stage lip SCC; no conclusions can be drawn for modern external beam techniques such as IMRT. The limited, brachytherapy-dominated evidence warrants cautious interpretation and justifies protocol standardisation, a core outcome set with validated quality-of-life instruments, and multi-institutional prospective registries.

## 1. Introduction

Cancer of the lip and oral cavity is among the most common malignancies worldwide, with approximately 400,000 new cases estimated in 2022 [1]; analyses of global trends over the period 1990–2021 indicate the persistence of this disease burden, with substantial socio-demographic disparities in its incidence and mortality [2]. At these sites, squamous cell carcinoma (SCC) is the predominant histological type, accounting for almost 90% of all lip and oral-cavity cancers [3,4]. Lip cancer chiefly affects the lower lip and is significantly associated with chronic exposure to ultraviolet radiation, tobacco and alcohol use, and a markedly higher incidence in men than in women [5]. Despite its relatively low frequency compared with other head and neck malignancies, this site is distinguished by a generally favourable prognosis in early stages, with five-year survival rates that may exceed 85–90% in published series [6].

The therapeutic management of lip SCC comprises surgery, external beam radiation therapy (EBRT) and brachytherapy (BT), used with either definitive or adjuvant postoperative intent; the choice of modality is dictated by tumour size and location, regional nodal status, available institutional resources, and the patient’s functional and aesthetic expectations [6]. Current international guidelines converge on this individualised approach: the NCCN guideline (Head and Neck Cancers, v.1.2026) recommends surgery as the preferred option in early-stage lip cancer, with definitive radiotherapy (including brachytherapy) as a curative alternative [7], whereas the EHNS–ESMO–ESTRO guideline emphasises the oncological equivalence of the two modalities in small tumours, with selection guided by functional and aesthetic outcome [8]. The available data suggest that both surgery and radiotherapy can achieve comparable local control in early stages, without either modality having demonstrated superiority in prospective randomised trials [9]. Indeed, in the absence of such trials systematically comparing treatment strategies, treatment selection remains, in practice, contingent upon institutional preferences and technological availability [6,9].

In this context, interstitial brachytherapy—both low dose rate (LDR) and high dose rate (HDR)—has been reported as an effective alternative to surgery, achieving local control rates of up to 94–97% in published series, accompanied by favourable late-toxicity profiles and satisfactory functional and aesthetic results [10]. External beam radiotherapy, including the modern IMRT and VMAT techniques, is used predominantly in the adjuvant postoperative setting, particularly in cases with risk factors such as positive resection margins or incomplete excision [11]. Consistent with the NCCN guideline, postoperative radiotherapy is indicated in the presence of adverse pathological features (positive margins, extranodal extension, pT3–pT4, pN2–pN3, perineural or vascular invasion), and, in the case of positive margins or extranodal extension, the concurrent addition of cisplatin-based chemotherapy is recommended [7,8]. Nevertheless, the considerable variability reported in the literature regarding doses, fractionation schedules, target-volume delineation and indications for nodal irradiation reflects the absence of a clear methodological consensus in the radiotherapeutic management of this site [9,10].

The rationale for adjuvant intensification rests on two landmark randomised trials—EORTC 22931 and RTOG 9501—whose combined analyses established that only positive resection margins and extracapsular nodal extension derive a significant benefit from the addition of concurrent cisplatin to postoperative radiotherapy [12,13,14]. From a staging perspective, the eighth edition of the AJCC/UICC system (2017) introduced depth of invasion into the definition of the T category for tumours of the lip and oral cavity, a parameter with a direct bearing on nodal risk and therapeutic decision-making; the temporal heterogeneity of the included studies, some of which predate the TNM era, nonetheless limits the comparability of staging [15].

The substantial global burden of this disease [16] contrasts with the absence, to date, of dedicated guidelines and of a specific systematic synthesis: although reviews exist on adjacent topics—for example, brachytherapy in early-stage oral-cavity cancers [10]—no dedicated systematic review, conducted in accordance with the PRISMA 2020 standards [17], has hitherto specifically addressed the reported practices, clinical outcomes and methodological variability of radiotherapy in squamous cell carcinoma of the lip as a distinct subsite; this observation is corroborated by the preliminary searches performed at registration in PROSPERO. Accordingly, the present systematic review, prospectively registered in PROSPERO (CRD420261349994), aims to synthesise the available evidence on the indications, technical modalities, doses, nodal management, toxicity and functional and aesthetic outcomes of radiotherapy in squamous cell carcinoma of the lip, and to identify the significant gaps in the current literature that warrant the direction of future research.

## 2. Materials and Methods

### 2.1. Protocol and Registration

The present systematic review was designed and conducted in accordance with the PRISMA 2020 guideline (Preferred Reporting Items for Systematic Reviews and Meta-Analyses) [17]. The protocol was prospectively registered in PROSPERO (the International Prospective Register of Systematic Reviews, maintained by the Centre for Reviews and Dissemination at the University of York, United Kingdom), before the commencement of data extraction, under the number CRD420261349994 (date of registration: 24 March 2026); the key registration fields are summarised in Supplementary Document S2, and the completed 27-item PRISMA 2020 [17] reporting checklist is provided in Supplementary Document S4. Prospective registration prevents the subsequent modification of eligibility criteria, objectives or analytical methods. Two deviations from the registered protocol arose during the conduct of the review and are transparently declared in Section 2.2. Ethics committee approval was not required, as the work is based exclusively on data from previously published studies. Generative artificial intelligence tools were used solely for language editing, for rendering the summary figures and tables from author-generated data, and for verifying bibliographic metadata; they were not used at any stage of the search, screening, data extraction, risk-of-bias assessment or interpretation. Full details are given in the Acknowledgments.

### 2.2. Eligibility Criteria

The eligibility criteria were pre-specified according to the PICO(S) framework registered in PROSPERO and are presented in full in Supplementary Document S1. The population of interest comprised adults aged at least 18 years with a histopathologically confirmed diagnosis of squamous cell carcinoma (SCC) of the lip, irrespective of subsite (lower lip, upper lip or oral commissure) and of TNM stage. Studies with mixed histology (SCC and basal cell carcinoma) were eligible only if the SCC-specific data could be reported or extracted separately; otherwise, such studies were excluded or included with explicitly declared reservations.

The intervention of interest encompassed all radiotherapy modalities: external beam radiotherapy (EBRT), irrespective of technique (orthovoltage, photons, IMRT, VMAT); and brachytherapy, irrespective of dose rate—LDR (low-dose-rate), HDR (high-dose-rate) or PDR (pulsed-dose-rate). Both definitive and adjuvant postoperative radiotherapy were eligible. PDR brachytherapy did not appear explicitly in the initial PROSPERO protocol and was subsequently deemed eligible on the basis of its radiobiological equivalence to continuous LDR, as recognised by the GEC-ESTRO recommendations [18]. This constitutes the first declared deviation from the registered protocol.

A comparator group was not mandatory; comparative data (for example, radiotherapy versus surgery) were extracted where available. Data on concurrent systemic chemotherapy were recorded contextually only, without constituting a central comparative axis, owing to the insufficiency of the available information for a meaningful synthesis. This represents the second declared deviation from the registered protocol.

The primary outcome measures were the local control rate (LC, %), disease-free survival (DFS, %) and overall survival (OS, %), reported at any evaluation timepoint. Secondary outcomes included acute and late radiotherapy-induced toxicity (assessed using any validated scale), description of the clinical target volume (CTV), the total doses and fractionation schedules used, elective nodal irradiation practices, and functional and aesthetic outcome measures. Eligible designs were randomised controlled trials, prospective cohorts, retrospective cohorts and case series of at least ten patients, published in English, with no date restriction.

### 2.3. Information Sources and Search Strategy

The systematic literature search was performed on 20 March 2026 across four sources: three bibliographic databases—PubMed/MEDLINE, the Cochrane Library (Trials Register) and Scopus—and one clinical-trials registry, ClinicalTrials.gov. Embase was not accessible to either reviewer, owing to the absence of an institutional subscription; this limitation was declared in the PROSPERO registration and is acknowledged in Section 4.8 of the manuscript. No restriction on publication date was applied, and the search covered the entire period from the inception of each source to the date of access. The search strategies combined controlled MeSH terms with free-text terms in the title and abstract fields, targeting lip neoplasms and all radiotherapy modalities; the full search strings are reproduced verbatim in Supplementary Document S3.

**Figure 1:**
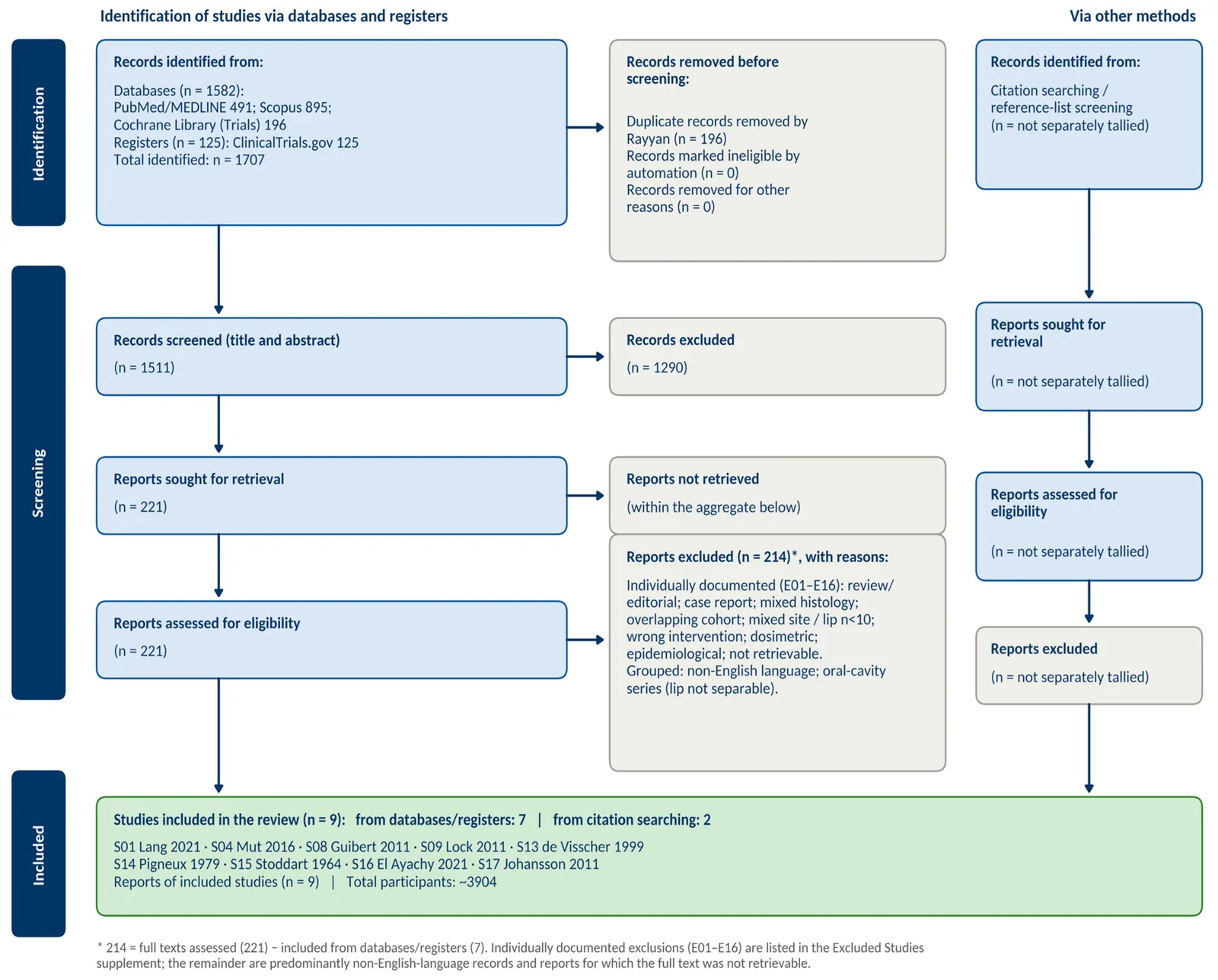
PRISMA 2020 flow diagram of study selection 22,23,24,25,26,27,28,29,30.

In addition to the electronic search, the reference lists of all included studies were hand-searched to identify eligible studies not captured by the initial search. Studies thus identified are recorded separately in the PRISMA flow diagram (Figure 1).

### 2.4. Study Selection

Records retrieved from the four sources were imported into the Rayyan platform (Rayyan Systems Inc., Cambridge, MA, USA; rayyan.ai) [19] for reference management and duplicate removal. After deduplication, two reviewers (O.C. and R.M.B.) independently examined the titles and abstracts of all remaining records, using the Blind Mode of the Rayyan platform, which masks each reviewer’s decisions until the assessment is complete. The full texts of the articles that passed this stage were independently assessed by the same two reviewers. Disagreements arising at any stage were resolved by discussion and consensus. The eligibility criteria were applied consistently across both stages. Studies removed at the full-text assessment stage, together with the reasons for exclusion, are presented in Supplementary Document S6 and in the PRISMA flow diagram (Figure 1).

### 2.5. Data Extraction

Data extraction was carried out using a pre-specified form, structured into 49 fields, developed in Microsoft Excel for Mac, version 16.89 (Microsoft Corporation, Redmond, WA, USA), before the commencement of extraction; the extraction methodology and the full list of data items are detailed in Supplementary Document S5. The form captured: study identification, design type, population characteristics (number of patients, age, sex distribution, subsite, TNM staging, histological confirmation), prior treatment, characteristics of the radiotherapy intervention (modality, intent, technique, total dose, fractionation, radiation source), target-volume description and elective nodal irradiation, concurrent systemic therapy, primary and secondary outcome measures with their evaluation timepoints and calculation methods, duration of follow-up, acute and late toxicity (scale used, grade distribution), and functional and aesthetic outcomes. Data were extracted by the first author (O.C.) and independently verified by the second author (R.M.B.). Fields for which information was not reported in the primary source were explicitly marked as ‘NR’ (not reported). No missing data were estimated or interpolated.

### 2.6. Risk-of-Bias Assessment

The risk of bias was assessed individually, for each included study, using the ROBINS-I tool (Risk Of Bias In Non-randomised Studies of Interventions) [20], chosen in keeping with the non-randomised nature of the entire body of identified studies; no randomised controlled trial met the eligibility criteria. ROBINS-I evaluates seven domains: (1) confounding, (2) selection of participants, (3) classification of interventions, (4) deviations from intended interventions, (5) missing data, (6) measurement of outcomes and (7) selective reporting of results. Each domain was assigned a rating of low, moderate, serious or critical risk; the overall assessment of each study reflected the most severe individual rating. The two reviewers (O.C. and R.M.B.) independently assessed all included studies, and disagreements were resolved by consensus. The results are reported in Section 3.6.

In keeping with the narrative synthesis (SWiM) of heterogeneous—predominantly single-arm—studies, and consistent with the registered protocol, the certainty of the body of evidence was not summarised through a formal GRADE Summary-of-Findings rating. Because the review yielded no pooled or comparative effect estimate—the included studies reporting single-arm proportions with heterogeneous outcome definitions and evaluation timepoints—a domain-based GRADE rating of an effect estimate would not have been meaningful and could have conveyed false precision. Certainty was therefore appraised structurally, through a per-study ROBINS-I assessment (Section 3.6) and a transparent narrative account; the exclusively observational, retrospective and small-sample nature of all included studies implies that the certainty of the available evidence is inherently limited, which is acknowledged among the limitations of the review (Section 4.8). The RoB-2 tool had been pre-specified for any randomised trials; as no randomised controlled trial met the eligibility criteria, only ROBINS-I was applied.

### 2.7. Data Synthesis

The substantial clinical and methodological heterogeneity among the included studies—spanning six decades of radiotherapy practice (1964–2021), diverse treatment modalities, distinct patient populations and heterogeneous outcome-reporting methods—precluded the statistical pooling of data. The synthesis was therefore conducted narratively, in accordance with the SWiM (Synthesis Without Meta-analysis) reporting framework [21]. The data were organised along five pre-specified thematic axes: (1) reported radiotherapy practices and their variability (modality, intent, dose, fractionation); (2) nodal management and elective nodal irradiation; (3) oncological outcome measures (local control, survival); (4) toxicity and functional and aesthetic outcomes; and (5) the identified gaps in consensus. Categorical conclusions were avoided where the available data did not support sufficient certainty. The results of this synthesis are presented in the following section, accompanied by summary tables and figures.

## 3. Results

### 3.1. Included Studies and General Characteristics

The selection process led to the inclusion of nine studies, of which seven were identified through the database search and two—El Ayachy 2021 [22] and Johansson 2011 [23]—through the hand-searching of reference lists (Figure 1). The nine studies comprised a total of approximately 3904 patients and spanned a broad time interval, from 1964 to 2021, originating from six countries (Germany, Spain, France, Canada, The Netherlands and Sweden). It should be noted that the figure of 3904 patients reflects the total cohorts of the included studies; the strict subset of squamous cell carcinoma of the lip that is actually analysable is approximately 3819 patients, the difference arising from studies with mixed histology or subsites (for example, Guibert 2011 [24], 92/172, and Johansson 2011 [23], 38/43). All studies were of a non-randomised design: seven retrospective cohorts—one of which had a comparative structure, contrasting radiotherapy with surgery (de Visscher 1999 [25])—and two case series of a historical character (Pigneux 1979 [26], Stoddart 1964 [27]). The full text was obtained for seven studies, whereas for one, only the abstract was available (de Visscher 1999 [25]), and for another, only partial text (Pigneux 1979 [26]).

In terms of population, the tumours were located predominantly on the lower lip, with most studies including early stages (T1–T2) and node-negative disease (N0). An exception was the Lang 2021 cohort [28], comprising advanced cases resected with positive margins (R1 or R2) and with nodal involvement (N+) in 14 of the 19 patients. Where reported, the median age was around 73–74 years (Guibert 2011 [24], El Ayachy 2021 [22], Johansson 2011 [23]), with a male predominance in the studies that recorded sex distribution. Cohort size varied considerably, from 19 patients (Lang 2021 [28]) to 3166 patients in the aggregated historical series of Stoddart 1964 [27]. The detailed study characteristics are presented in Table 1, and the complete selection flow in Figure 1.

### 3.2. Reported Radiotherapy Practices and Their Variability

The irradiation modalities used were heterogeneous. External beam radiotherapy (EBRT) as the sole modality was reported in a single study (Lang 2021 [28]), in the adjuvant setting, using the IMRT (15/19 patients) and 3D-CRT (4/19) techniques. Brachytherapy was the dominant modality: high-dose-rate (HDR) in one study (Mut 2016 [29]), low-dose-rate (LDR) in three studies (Guibert 2011 [24], Lock 2011 [30], Pigneux 1979 [26]) and pulsed-dose-rate (PDR) in two studies (El Ayachy 2021 [22], Johansson 2011 [23]). One historical study combined orthovoltage external radiotherapy with LDR interstitial brachytherapy (Stoddart 1964 [27]), and in one study the irradiation modality was not reported (de Visscher 1999 [25]).

With the exception of Lang 2021 [28], in which the intent was adjuvant, all other studies used radiotherapy with definitive intent, some of them including both primarily treated and postoperative cases (Guibert 2011 [24], El Ayachy 2021 [22], Johansson 2011 [23]).

Total doses and fractionation schedules varied by modality. In HDR brachytherapy, Mut 2016 [29] delivered 45 Gy in nine fractions, twice daily (5 Gy/fraction). In LDR brachytherapy, Guibert 2011 [24] reported a mean dose of 65 Gy (range 55–77 Gy), delivered continuously, whereas Lock 2011 [30] employed a particular technique using permanent radioactive gold-grain implants (Au-198), at a dose of 55 Gy. In PDR brachytherapy, median doses were 70.14 Gy (range 60–85 Gy; El Ayachy 2021 [22]) and 60 Gy (range 55–66 Gy; Johansson 2011 [23]), delivered in pulses of 0.83 Gy at two-hour intervals. In the study with adjuvant external radiotherapy, the dose to the primary tumour was 66 Gy (range 60–70 Gy) and that to the cervical nodes was 54 Gy (range 50–60 Gy), in conventional fractionation (Lang 2021 [28]). These values are concordant with the NCCN recommendations for the postoperative radiotherapy of risk features (60–66 Gy at 2 Gy/fraction) and with boost-type brachytherapy schedules (LDR 20–35 Gy or HDR 21 Gy in combination with 40–50 Gy EBRT) [7]. For the historical studies (Pigneux 1979 [26], Stoddart 1964 [27]), doses were not reported, and in the abstract-only study (de Visscher 1999 [25]) the modality, dose and technique remained unreported. Details of doses and fractionation are summarised in Table 2.

### 3.3. Nodal Management and Elective Nodal Irradiation

Elective nodal irradiation was practised in a single study, Lang 2021 [28], in which 18 of the 19 patients (94.7%) had cervical nodal irradiation included in the treatment plan, at a dose of 54 Gy—an approach correlated with the presence of nodal involvement (N+) in this cohort. In the remaining studies, comprising predominantly N0 populations, elective nodal irradiation was not performed. Guibert 2011 [24] explicitly recorded a ‘wait-and-see’ policy, without prophylactic nodal treatment, with a nodal recurrence rate of 5.4% (5 of 92). Johansson 2011 [23] reported regional control of 93% (three regional recurrences of 43), and El Ayachy 2021 [22] noted, in its conclusions, a recommendation for a multimodal approach to regional control, without specifying the elective irradiation practice (NR). This expectant approach in N0 tumours is consistent with the NCCN guideline, which states that elective neck dissection or irradiation is not generally indicated in mucosal lip carcinoma at stages T1–T3 N0 [7]. In the other studies, nodal management was not reported.

### 3.4. Oncological Outcome Measures (Local Control and Survival)

**Figure 2:**
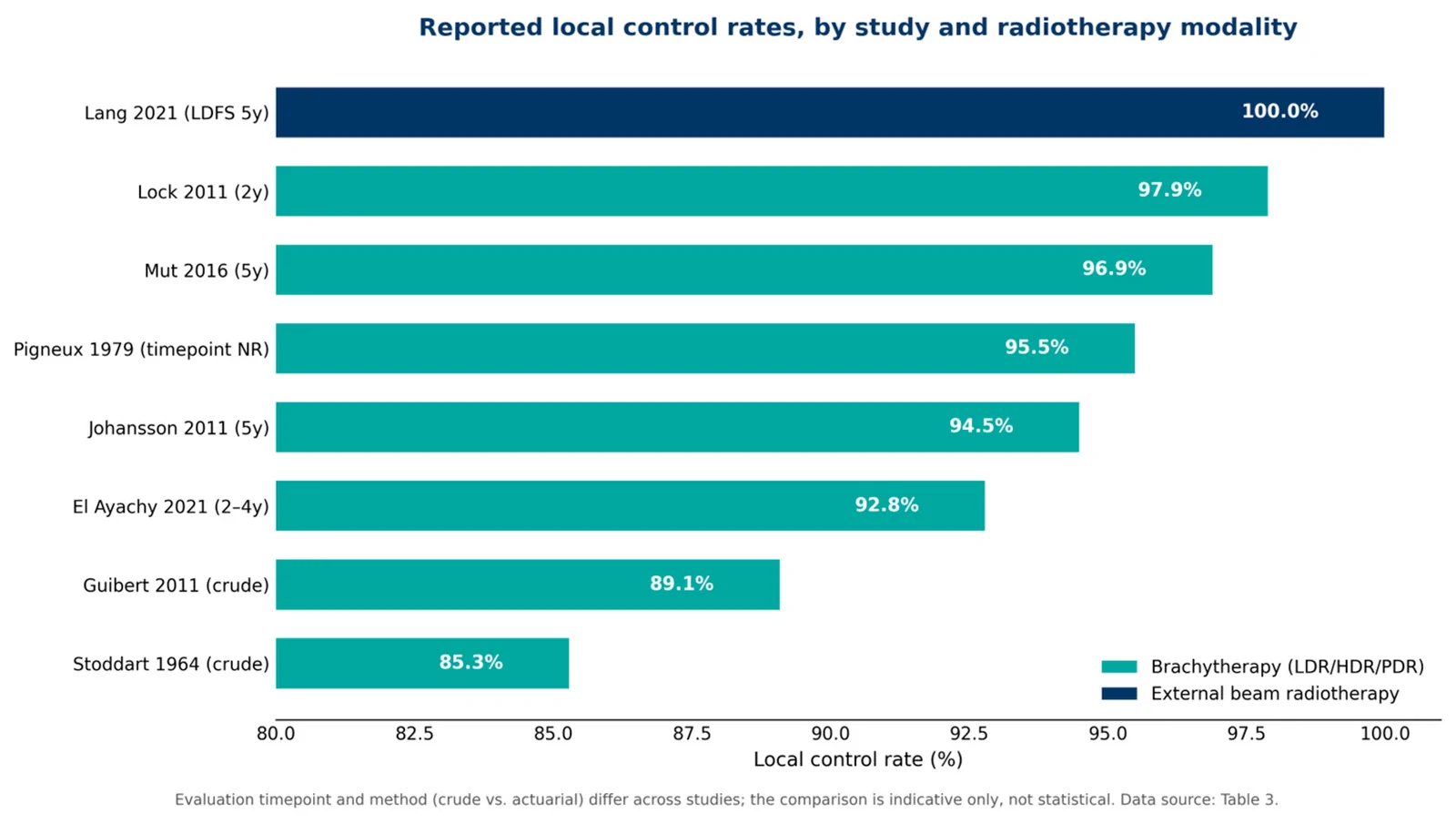
Reported local control rates, by study and radiotherapy modality [22,23,24,26,27,28,29,30]. The evaluation timepoint and method (crude vs. actuarial) differ across studies; the comparison is indicative only, not statistical. Data source: Table 3.

Local control was the most frequently reported measure, being documented, in one form or another, in all nine studies, although the calculation methods and evaluation timepoints were heterogeneous (Table 3; Figure 2). Local control rates ranged from 85.3% (the historical series of Stoddart 1964 [27], crude calculation) to 100% (Lang 2021 [28], expressed as local recurrence-free survival at 5 years). In the brachytherapy studies, local control was generally high: 96.9% at 5 years for HDR (Mut 2016 [29], actuarial calculation; 100% for T1 and 93.2% for T2), 89.1% for LDR (Guibert 2011 [24], crude calculation, 10 recurrences of 92), 97.9% at 2 years for LDR with permanent implants (Lock 2011 [30]), and 95.5% in a historical LDR study (Pigneux 1979 [26], timepoint not reported). In PDR brachytherapy, local control was 92.8% at 2 and 4 years (El Ayachy 2021 [22], actuarial calculation), 97.6% at 2 years, 94.5% at 5 years and 94.5% at 10 years (Johansson 2011 [23], actuarial calculation). The comparative study de Visscher 1999 [25] reported local control ‘comparable’ to that of surgery, without stating the exact value (NR).

When the corpus is stratified by therapeutic era, the apparent homogeneity of these figures dissolves. Two historical series employed pre-1980s techniques—orthovoltage external radiotherapy combined with radium-based LDR brachytherapy (Stoddart 1964 [27]) and early LDR interstitial brachytherapy (Pigneux 1979 [26])—and reported crude or incompletely specified local control of 85.3% and 95.5%, respectively, with no survival, toxicity or functional data. The six modern series (published 2011–2021, employing iridium-192 LDR/PDR/HDR brachytherapy, permanent gold-grain (Au-198) implants or IMRT/3D-CRT) reported local control ranging from 89.1% (crude; Guibert 2011 [24]) to 97.9% (2-year; Lock 2011 [30]), with the actuarial (Kaplan–Meier) estimates clustering between 92.8% and 96.9% (El Ayachy 2021 [22], Johansson 2011 [23], Mut 2016 [29]) and the single adjuvant cohort reporting 100% local disease-free survival at 5 years (Lang 2021 [28]); these series also reported survival, toxicity and, in part, functional outcomes. The intermediate, abstract-only study (de Visscher 1999 [25]) could not be assigned to either stratum, as its modality and technique were not reported. Consequently, the frequently cited range of approximately 93–97% derives exclusively from the modern actuarial series and must not be read as an aggregate estimate of the entire corpus. This temporal stratification should itself be interpreted with caution, as era is confounded with reporting quality and with the outcome-calculation method (crude versus actuarial), so that even the stratified comparison remains descriptive and hypothesis-generating only.

**Figure 3:**
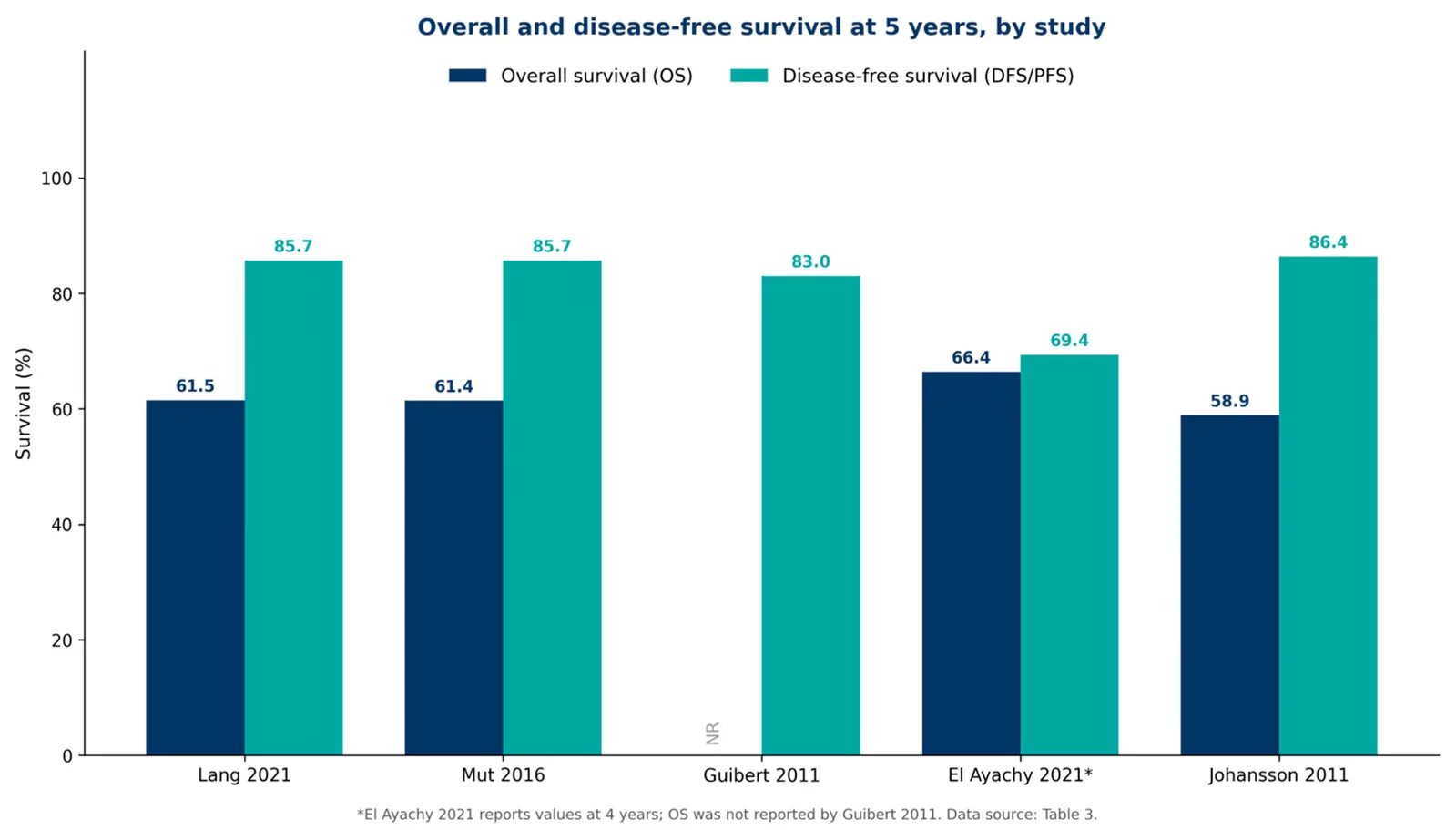
Overall survival (OS) and disease-free survival (DFS/PFS) at 5 years, by study [22,23,24,28,29]. * El Ayachy 2021 [22] reports values at 4 years; OS was not reported by Guibert 2011 [24]. Data source: Table 3.

Disease-free survival (DFS/RFS) was reported in five studies, with values of 85.7% at 5 years (Lang 2021 [28], expressed as progression-free survival; Mut 2016 [29]), 83% at 5 years (Guibert 2011 [24], with 85% at 2 years and 75% at 8 years), 69.4% at 4 years (El Ayachy 2021 [22], progression-free survival) and 86.4% at 5 and 10 years (Johansson 2011 [23], with 92.7% at 2 years). It must be emphasised, from a methodological standpoint, that these measures are not equivalent: disease-free survival (DFS), recurrence-free survival (RFS) and progression-free survival (PFS) have distinct definitions, and their descriptive juxtaposition was undertaken solely for indicative comparison, not as a single measure. Overall survival (OS) was reported in only four studies, with five-year values ranging between 58.9% (Johansson 2011 [23]) and 61.5% (Lang 2021 [28])—61.4% for Mut 2016 [29]—and 66.4% at 4 years (El Ayachy 2021 [22], a different evaluation timepoint, at 4 years, flagged as such); in the other studies, overall survival was not reported. The median duration of follow-up, where recorded, ranged from 27 months (Lock 2011 [30]) to approximately 65 months (Guibert 2011 [24]). The measures are presented comparatively in Table 3 and Figure 3.

### 3.5. Toxicity and Functional and Aesthetic Outcomes

Acute toxicity was reported in five studies, using heterogeneous scales (CTCAE v3.0, CTCAE v4, LENT-SOMA or non-standardised clinical assessment). The dominant acute reaction was mucositis: Mut 2016 [29] reported grade ≥3 mucositis in all patients (LENT-SOMA), resolving within a few weeks; El Ayachy 2021 [22] recorded grade ≥ 2 radiomucositis in all patients, and Johansson 2011 [23] intense mucositis in all patients, clinically assessed without a formal scale—in both studies with early onset and remission within approximately four weeks; Lang 2021 [28] reported frequent grade 1–2 mucositis and xerostomia, with grade 3 forms in a minority (exact proportion not reported); and Guibert 2011 [24] recorded no grade 3/4 toxicity, with side effects of any grade in 16.3% of patients. In the historical and abstract-only studies (Pigneux 1979 [26], Stoddart 1964 [27], de Visscher 1999 [25]), acute toxicity was not reported.

Late toxicity was reported in six studies. Most recorded no grade 3/4 late toxicity (Lang 2021, Mut 2016 [29], Guibert 2011 [24]). El Ayachy 2021 [22] reported grade 2 fibrosis in three patients (8.3%) and grade 2 chronic pain in one patient, with no toxicity of grade 3 or higher. Johansson 2011 [23] reported one case of soft-tissue necrosis (2%) and one case of osteoradionecrosis (2%, confounded with prior irradiation of the tongue), together with salivary leakage and lip stiffness in six patients (14%) and periodontitis in three patients (7%). Lock 2011 [30], in which cosmesis was the primary objective, reported ‘good’ results, with no grade 3 toxicity described.

Functional or aesthetic outcomes were reported in six studies, using predominantly non-standardised methods. Cosmetic outcomes were rated ‘good’ or ‘satisfactory’ in high proportions: 94% (Mut 2016 [29]), 92% (Guibert 2011 [24], with satisfactory functional outcomes in 99% of patients) and ‘generally very good’ (Johansson 2011 [23]). A single study used a validated quality-of-life instrument (El Ayachy 2021 [22]), reporting, through the FACT-H&N questionnaire, a median total score of 127 of 148 and a Trial Outcome Index of 84, with all patients stating that they would recommend the treatment. In the historical and abstract-only studies, functional and aesthetic outcomes were not reported. Data on toxicity and functional outcomes are summarised in Table 3.

### 3.6. Results of the Risk-of-Bias Assessment

**Figure 4:**
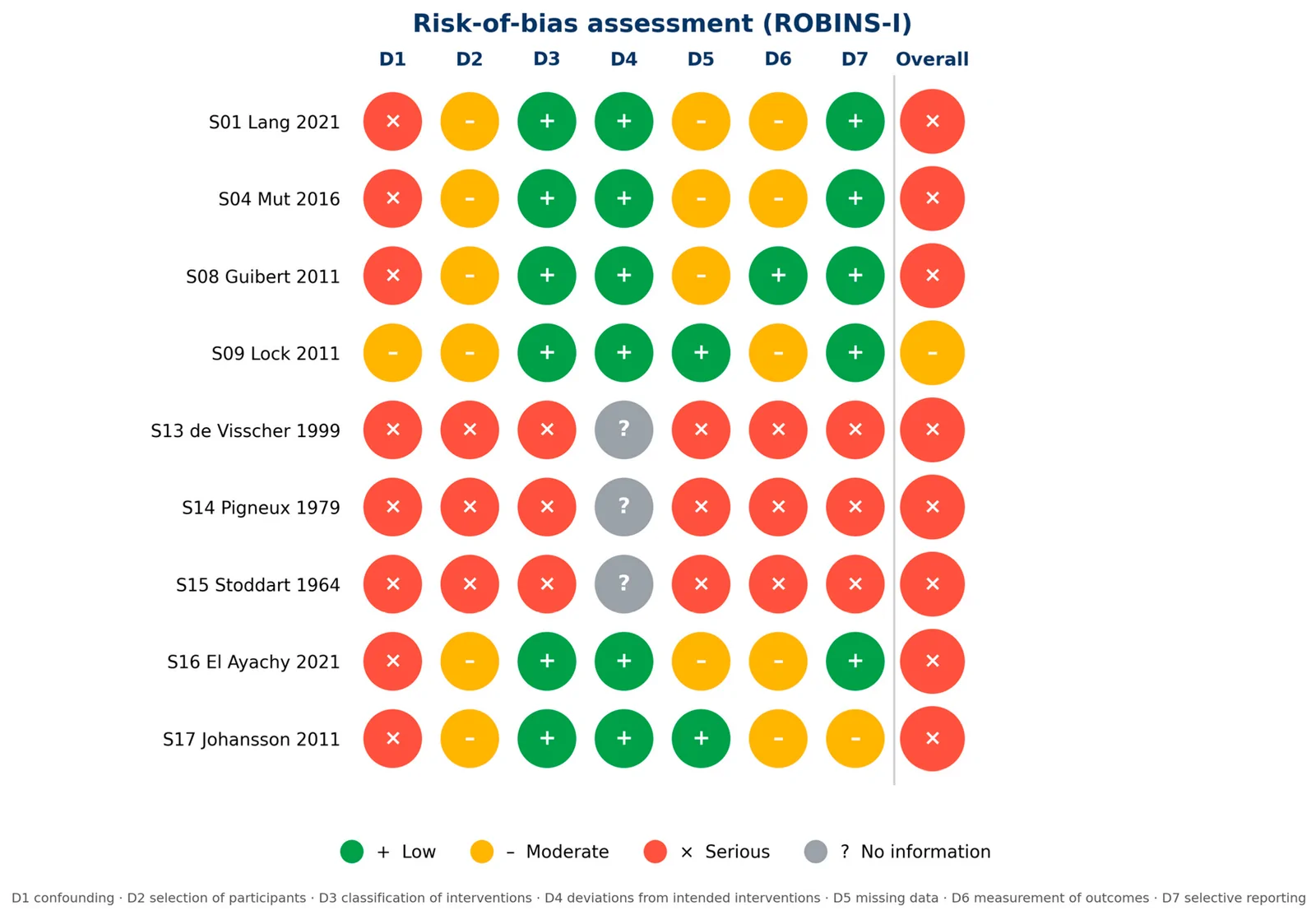
Risk-of-bias assessment (ROBINS-I) for the nine included studies [22,23,24,25,26,27,28,29,30], across the seven domains and the overall assessment (‘traffic-light’ representation). Green = low risk; yellow = moderate; red = serious; grey = no information.

The risk-of-bias assessment using the ROBINS-I tool classified eight of the nine studies in the serious-risk category, with a single study (Lock 2011 [30]) rated at moderate risk—the lowest level across the entire body of studies. The domain most frequently responsible for the serious rating was confounding, reflecting the retrospective design, the absence of a comparator arm and, in some cases, the mixed histology or the combination of primary with recurrent or postoperative cases (Guibert 2011 [24], El Ayachy 2021 [22], Johansson 2011 [23]). The abstract-only study (de Visscher 1999 [25]) and the two historical studies (Pigneux 1979 [26], Stoddart 1964 [27]) received a serious rating across all domains, owing to insufficient information on modality, dose and outcome reporting. The detailed results across the seven domains are presented in Table 4 and, in summary form, in Figure 4; the distribution of the risk of bias across domains is shown in Figure S2 and an evidence map of the included studies in Figure S3 (Supplementary Document S7). In detail, the ‘serious’ ratings were driven by the following domain-level judgements: in the confounding domain (D1), by the absence of any adjustment for baseline prognostic factors (stage, subsite, prior treatment) in uncontrolled single-arm designs; in the participant-selection domain (D2), by retrospective, single-institution recruitment over long intervals without safeguards for consecutive sampling; in the missing-data domain (D5), by incompletely reported losses to follow-up; and in the outcome-measurement domain (D6), by unblinded, clinician-assessed endpoints and heterogeneous or non-standardised toxicity scales. For the two historical series and the abstract-only study, domains D3–D7 could frequently not be judged at all and were rated ‘serious’ or ‘no information’ accordingly.

## 4. Discussion

### 4.1. Summary of Principal Findings

The present systematic review brought together nine studies, totalling approximately 3904 patients treated with radiotherapy for squamous cell carcinoma of the lip, over six decades (1964–2021) and across six countries. The narrative synthesis highlighted two principal, seemingly complementary findings: on the one hand, radiotherapy—and, in particular, brachytherapy—appears to offer high local control rates in squamous cell carcinoma of the lip, with favourable toxicity profiles and good aesthetic and functional outcomes; on the other, the literature is marked by considerable methodological variability and by the absence of consensus regarding doses, fractionation, target-volume delineation and nodal management. These two equally essential themes structure the discussion that follows. Given that seven of the nine included studies evaluated brachytherapy, these findings should be read, from the outset, as describing primarily the outcomes of brachytherapy—much of it historical—rather than of radiotherapy in general (Section 4.2 and Section 4.7).

### 4.2. Brachytherapy as an Effective and Conservative Alternative

Brachytherapy was the dominant modality in the identified literature, reported in seven of the nine included studies, in all three dose-rate forms—LDR, HDR and PDR. The reported local control rates were consistently high. In the brachytherapy studies with actuarial (Kaplan–Meier) data, local control was generally between approximately 93% and 97% (Mut, 96.9% at 5 years [29]; El Ayachy, 92.8% at 2–4 years [22]; Johansson, 94.5% at 5 and 10 years [23]). This range derives exclusively from the modern actuarial series; the two historical series reported crude or incompletely specified estimates of 85.3% and 95.5%, which are not directly comparable and are presented separately in Section 3.4. Lock reported local control of 97.9% at two years [30], and Guibert a crude local control of 89.1% [24]. Aesthetic and functional outcomes were rated good or very good in most series. These observations are consistent with the position expressed in the GEC-ESTRO recommendations for the brachytherapy of head and neck squamous cell carcinomas [18,31] and in the ACROP recommendations for skin brachytherapy [32], which recognise interstitial brachytherapy as a curative, organ-preserving option in lip tumours. They are also coherent with the conclusions of the systematic review by Draghini and colleagues, according to which interventional brachytherapy may represent an alternative to surgery in early-stage oral-cavity cancers [10], and with the SCRiBE meta-analysis, which compared brachytherapy with external radiotherapy in cutaneous cancers [33]. Taken together, the synthesised data support the notion that lip brachytherapy, applied in experienced centres, is associated with durable tumour control and low morbidity.

A particular aspect is represented by the study of Lock and colleagues, the only one in the body of studies rated at moderate risk of bias by the ROBINS-I tool; it reported a local control rate of 97.9% at two years using a permanent-implant technique with radioactive gold grains (Au-198), a distinct technical approach with the advantage of a minimally invasive procedure [30]. In turn, the two PDR brachytherapy studies made complementary contributions: El Ayachy and colleagues were the only investigators to assess quality of life using a validated instrument (FACT-H&N), obtaining high scores [22], and Johansson and colleagues reported outcomes over the longest time horizon in the entire corpus, with an actuarial local control rate of 94.5% maintained at ten years [23]. These results, although derived from small, retrospective series, suggest durability of local control and a favourable long-term tolerance.

For comparative context, surgical series of early-stage lip carcinoma report, in their turn, local control rates exceeding 90%, which supports the notion of oncological equivalence between the two treatment strategies and shifts the criterion of choice towards morbidity and functional and aesthetic outcome, rather than towards oncological efficacy per se [9].

### 4.3. Variability of Practice and Absence of Consensus

In contrast with the relative consistency of the oncological outcomes, the reported radiotherapy practices were marked by pronounced heterogeneity. Total doses ranged from 45 Gy in HDR brachytherapy [29] to median values of approximately 70 Gy in PDR brachytherapy [22], with fundamentally different fractionation schedules between modalities, and doses were not reported at all in the historical studies. The choice of modality reflected tradition and institutional resources rather than uniform criteria: external radiotherapy, used as the sole modality, appeared exclusively in the adjuvant setting, in advanced cases or those with positive resection margins [28], whereas brachytherapy was reserved for early tumours treated with definitive intent. This division of roles is, in itself, clinically reasonable; nonetheless, the absence of randomised trials directly comparing the modalities—including the comparison of HDR with LDR, addressed only outside our corpus [34]—leaves open the question of the optimal schedule. The observed variability confirms the need for the standardisation of protocols, as also signalled by the updated GEC-ESTRO recommendations [31].

From a radiobiological standpoint, the direct comparison of schedules with fundamentally different dose rates and fractionations (for example, 45 Gy HDR in nine fractions versus 70 Gy PDR) is imprecise in the absence of normalisation to the equivalent dose in fractions of 2 Gy (EQD2) using the linear-quadratic model; the use of an α/β ratio of approximately 10 Gy for the tumour effect of squamous cell carcinomas would allow a more rigorous comparison of the reported schedules and would reduce the risk of conflating differences in physical dose with genuine differences in efficacy [18].

### 4.4. Nodal Management

Nodal management constituted a particularly heterogeneous and insufficiently documented domain. Elective nodal irradiation was practised in a single study, that of Lang and colleagues, in which it was applied to 94.7% of patients, consistent with the presence of nodal involvement in a cohort with advanced disease [28]. In node-negative populations, the approach was generally expectant, of the ‘wait-and-see’ type, with low regional recurrence rates [23,24]. This conduct is supported by the NCCN guideline, which does not recommend, as a rule, the elective treatment of the neck in mucosal lip carcinoma at stages T1–T3 N0 [7]; the EHNS–ESMO–ESTRO guideline, however, conditions the decision upon risk factors such as depth of invasion, which highlights precisely the gap in uniform criteria in the analysed literature [8]. Although these data suggest that, in early-stage N0 disease, the omission of prophylactic nodal treatment may be reasonable, the absence of explicit and uniform criteria for selecting patients for elective irradiation remains an important gap in the literature.

From the perspective of clinical decision-making, the rate of occult nodal metastases in lip carcinoma is low in early stages but rises progressively with T category, with depth of invasion and with the degree of dedifferentiation; according to the classic decision principle of Weiss and colleagues, elective irradiation or dissection of the clinically negative neck becomes justified when the probability of occult involvement exceeds approximately 20% [35]. The application of such a quantitative threshold, correlated with the depth of invasion from the eighth-edition AJCC staging [15], would provide precisely the objective selection criterion that is lacking in the analysed studies.

### 4.5. Concurrent Systemic Therapy

One aspect that merits emphasis, precisely through its absence, is the role of concurrent systemic therapy. A single study documented the administration of cisplatin, and that was on a purely contextual basis, in an adjuvant cohort with risk factors [28]; no study comparatively evaluated chemoradiotherapy versus radiotherapy alone. Although the NCCN and EHNS–ESMO–ESTRO guidelines recommend the addition of cisplatin to postoperative radiotherapy in the presence of positive margins or extranodal extension—a situation encountered in the Lang cohort, with R1/R2 resections [7,8]—the analysed corpus does not permit a comparative evaluation of this strategy in squamous cell carcinoma of the lip. Consequently, the contribution of concurrent chemotherapy at this site remains, at present, an evidence gap rather than a data-supported practice. We have therefore treated this aspect exclusively as a direction for research, avoiding any comparative conclusion, in keeping with the prospectively registered protocol.

At the level of head and neck squamous cell carcinomas as a whole, the MACH-NC meta-analysis demonstrated an absolute overall-survival benefit of approximately 6.5% at 5 years from the addition of concurrent chemotherapy to radiotherapy [36], and the combined analysis of the EORTC 22931 and RTOG 9501 trials restricted this benefit, in the postoperative setting, to the subgroup with positive resection margins or extracapsular extension [14]. The extrapolation of these data to the lip site—usually diagnosed at early stages and with a favourable prognosis—must be undertaken with caution, in the absence of dedicated studies, which reaffirms the status of chemoradiotherapy at this site as a direction for research rather than a therapeutic standard.

### 4.6. Heterogeneity of Outcome Reporting

The synthesis was further limited by the heterogeneity of outcome reporting. Although local control was documented—in one form or another—in all studies, overall survival was reported in only four of them, and disease-free survival in five. Toxicity was assessed using different scales (CTCAE in various versions, LENT-SOMA or non-standardised clinical assessment), which precluded any direct comparison of morbidity profiles. Notably, only a single study used a validated quality-of-life instrument [22]. This variability in reporting, together with the differences in evaluation timepoints and calculation methods (crude versus actuarial), rendered statistical pooling impossible and mandated an exclusively narrative synthesis, in accordance with the SWiM framework [21].

For this reason, the adoption of standardised outcome sets (core outcome sets) and the systematic use of validated quality-of-life instruments—such as the EORTC QLQ-C30 together with the site-specific H&N35 module, alongside the FACT-H&N [22]—would considerably enhance the comparability between future studies. It must be stated plainly, however, that a statistical adjustment for confounding based on individual patient data (IPD) is not feasible with the currently available literature, which consists almost exclusively of retrospective single-arm series without accessible patient-level data; only prospectively collected, standardised data can remove this barrier.

### 4.7. Quality of the Evidence and Risk of Bias

The methodological quality of the body of studies must be interpreted with caution. Eight of the nine studies were rated at serious risk of bias using the ROBINS-I tool, with a single study rated as moderate [30]. The domain most frequently affected was confounding, a predictable consequence of the retrospective, single-centre design—lacking a comparator arm—of most studies. The historical and abstract-only studies received a serious rating across all domains, owing to insufficient information. Consequently, although the oncological outcomes are encouraging and consistent, they are derived from a body of evidence of limited certainty, and the conclusions must be framed with the corresponding reservation. A further caveat concerns the representativeness of this evidence with respect to contemporary practice: seven of the nine included studies evaluated brachytherapy; a single study evaluated external beam radiotherapy alone, and intensity-modulated radiotherapy (IMRT) was represented only within that study, in 15 of its 19 patients—a small, highly selected postoperative cohort that cannot support any inference about IMRT at this site. The present review therefore characterises, in effect, the outcomes of largely historical brachytherapy rather than of ‘radiotherapy in general’, and no conclusion regarding the efficacy of IMRT—currently the mainstream external beam technique in head and neck cancer—can be drawn from this body of evidence. The correspondence between the principal findings of the review and the recommendations of the major international guidelines (NCCN, GEC-ESTRO, EHNS–ESMO–ESTRO) is summarised in Table 5.

### 4.8. Strengths and Limitations of the Review

This review has several strengths. It was prospectively registered in PROSPERO before data extraction, was conducted in accordance with the PRISMA 2020 guideline [17], with two independent reviewers working in the Blind Mode of the Rayyan platform [19], and included a rigorous risk-of-bias assessment using ROBINS-I [20], as well as a structured synthesis in accordance with the SWiM framework [21]. All deviations from the protocol were transparently declared. There are, however, limitations that must be honestly acknowledged. The Embase database could not be accessed owing to the absence of an institutional subscription—a significant methodological shortcoming for a systematic review—and the eligibility criteria were restricted to English-language publications. Taken together, the exclusion of Embase and the English-language restriction create a high probability that significant, relevant studies—in particular European brachytherapy series published in other languages—have been overlooked, and the completeness of the present evidence base must be judged accordingly. The per-reason breakdown of exclusions at the full-text stage could not be reconstructed retrospectively from the screening platform and is reported in aggregate, in accordance with the PRISMA 2020 [17] recommendations. Furthermore, the inclusion of historical studies, originating from different therapeutic eras, and the heterogeneity of reporting mandated a narrative synthesis, without the possibility of a meta-analysis.

In addition, the small size of most cohorts raises the problem of small-study effects and publication bias; these could not be formally evaluated (for example, by funnel plots or asymmetry tests) owing to the insufficient number of homogeneous studies. Furthermore, consistent with the registered protocol and the narrative nature of the synthesis, the certainty of the evidence was not formally assessed; this is acknowledged as a limitation, and the conclusions should be read as descriptive rather than as graded recommendations.

### 4.9. Clinical Implications and Future Directions

**Figure 5:**
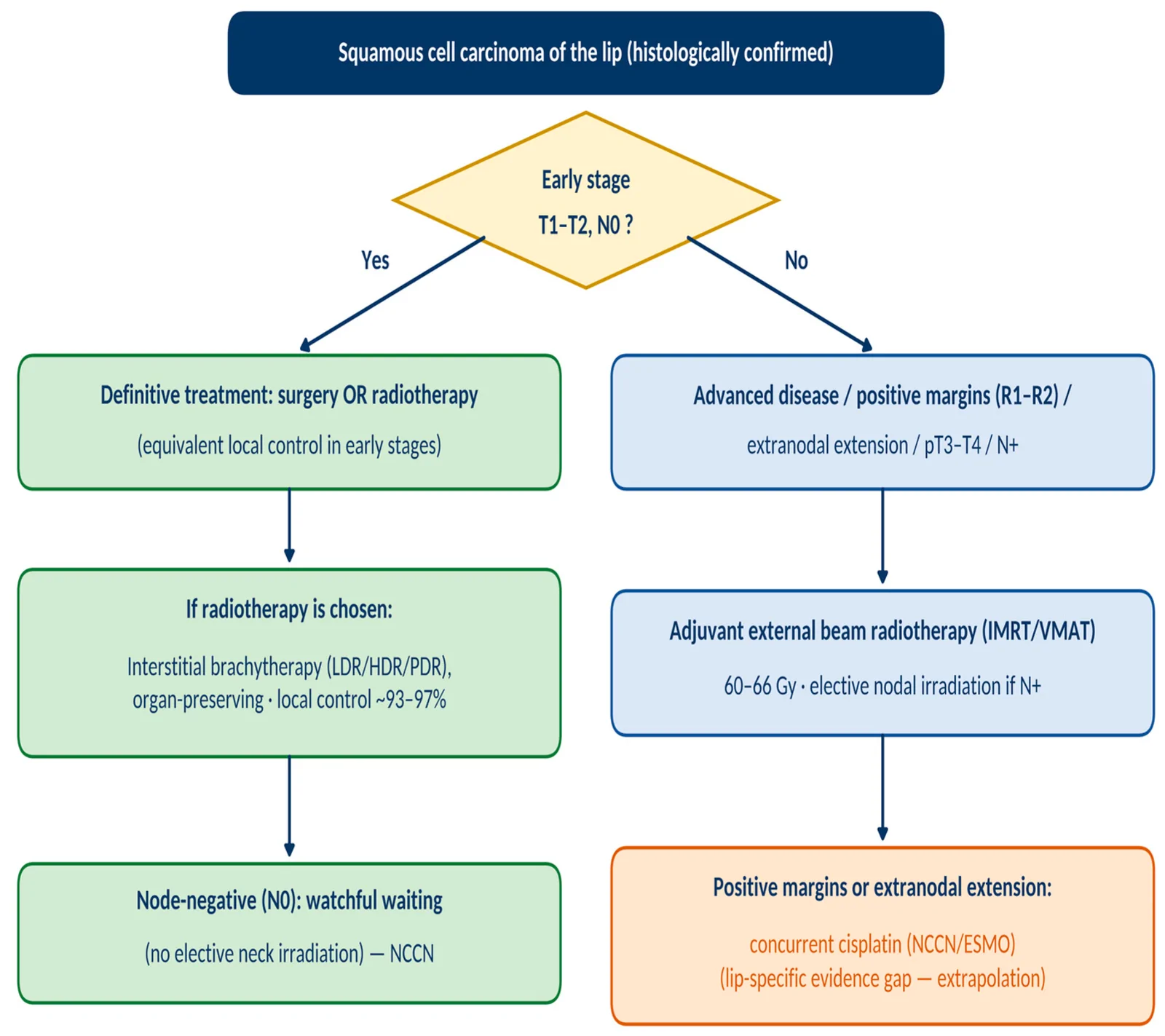
Proposed conceptual framework for selecting the radiotherapy modality in squamous cell carcinoma of the lip, synthesised from the included studies and international guidelines (NCCN; GEC-ESTRO; EHNS–ESMO–ESTRO). The framework is author-derived, has not been prospectively validated and is intended to support—not to direct—multidisciplinary tumour-board decision-making; the indication for concurrent cisplatin is extrapolated from head and neck cancer guidelines, with no lip-specific comparative evidence.

Notwithstanding these limitations, the present review offers a systematic synthesis, compliant with the PRISMA 2020 [17] standards, of the practices and outcomes of radiotherapy in squamous cell carcinoma of the lip. The data suggest that interstitial brachytherapy represents an effective and conservative option for early-stage tumours, whereas external beam radiotherapy retains a well-defined role in the adjuvant setting, in advanced cases; a synthesis of these indications, corroborated by the international guidelines, is presented in the form of a proposed conceptual framework (Figure 5), to be read as a hypothesis-generating synthesis rather than a validated decision tool. Future research directions follow directly from the identified gaps: the standardisation of doses, fractionation schedules and target-volume definitions; the elaboration of uniform criteria for elective nodal irradiation; the systematic adoption of validated quality-of-life instruments; and the clarification of the role of concurrent systemic therapy. Because retrospective statistical rescue of the existing literature—including adjustment based on individual patient data—is not feasible (Section 4.6), we explicitly advocate two concrete instruments: (i) the establishment of a multi-institutional prospective patient registry dedicated to lip carcinoma treated with radiotherapy, ideally built upon existing collaborative structures such as those coordinated by the GEC-ESTRO working group, and (ii) the development of a Core Outcome Set (COS) incorporating validated quality-of-life instruments, which would render future single-arm studies mutually comparable and prepare the ground for the methodological consensus that is currently lacking.

A particularly promising direction is sentinel-lymph-node biopsy, already integrated into the NCCN guideline for the oral cavity as a method for staging the clinically negative neck; its application in lip carcinoma could refine the selection of patients for nodal treatment, reducing both the risk of undertreatment and the morbidity of unnecessary dissections [7]. Equally, the integration of functional imaging and of staging based on depth of invasion (AJCC eighth edition) [15], together with the evaluation of molecular prognostic factors, could support a genuinely personalised approach to radiotherapy at this site.

## 5. Conclusions

The present systematic review, conducted in accordance with the PRISMA 2020 [17] standards and synthesised narratively using the SWiM framework, brought together nine studies and approximately 3904 patients treated with radiotherapy for squamous cell carcinoma of the lip. The available data—deriving, in seven of the nine studies, from interstitial brachytherapy, much of it historical—suggest that radiotherapy at this site is associated with high local control rates in early-stage disease, with favourable toxicity profiles and generally good functional and aesthetic outcomes. These observations support the role of brachytherapy as an effective, organ-preserving therapeutic option, whereas external beam radiotherapy retains a well-defined place in the adjuvant setting, in advanced cases or those with risk factors. Strictly speaking, these conclusions apply to the brachytherapy-dominated body of evidence identified; no inference can be drawn regarding modern high-precision external beam techniques such as IMRT, for which the only lip-specific data identified comprised a single 19-patient postoperative cohort.

At the same time, the review highlights considerable methodological variability and the absence of consensus regarding doses, fractionation schedules, target-volume delineation and nodal management, within a literature composed predominantly of retrospective studies at a risk of bias rated, in most cases, as serious; the conclusions must therefore be interpreted with the corresponding caution. The findings justify the need for efforts towards standardisation—of dose and fractionation protocols, of elective nodal irradiation criteria and of outcome reporting, through a dedicated core outcome set (COS) and validated quality-of-life instruments—as well as the development of multi-institutional prospective registries and collaborative initiatives that would enable the building of the methodological consensus currently lacking.

## Figures and Tables

**Table 1:** Characteristics of the nine included studies.

Study	Country	Design	N Total (Lip SCC)	Subsite	Stage (TNM)	Histology	ROBINS-I	RT Modality	Complications/QoL Reporting
S01 Lang 2021 [28]	Germany	Retrospective cohort	19	Lip (adjuvant)	Advanced; R1/R2; N+ 14/19	SCC	Serious	EBRT	Acute + late toxicity (graded); no validated QoL
S04 Mut 2016 [29]	Spain	Retrospective cohort	68	Lip	T1–T2 N0	SCC	Serious	BT-HDR	Toxicity (LENT-SOMA); cosmesis (non-validated)
S08 Guibert 2011 [24]	France	Retrospective cohort	92 (of 172)	Lip	T1–T2 N0 (early)	SCC (subset of mixed)	Serious	BT-LDR	Toxicity (graded); function/cosmesis (non-validated)
S09 Lock 2011 [30]	Canada	Retrospective cohort	51	Lip	Early	SCC	Moderate	BT-LDR	Late toxicity; cosmesis (non-validated)
S13 de Visscher 1999 [25]	The Netherlands	Retrospective comparative cohort (RT vs. surgery)	256	Lip	NR	SCC	Serious	NR	NR
S14 Pigneux 1979 [26]	France	Historical case series	91	Lip	NR	SCC	Serious	BT-LDR	NR
S15 Stoddart 1964 [27]	Canada	Historical case series	3166	Lip	NR	SCC	Serious	Mixed (EBRT + BT-LDR)	NR
S16 El Ayachy 2021 [22]	France	Retrospective cohort	38	Lip	Early–intermediate	SCC	Serious	BT-PDR	Toxicity (graded); QoL: FACT-H&N (validated)
S17 Johansson 2011 [23]	Sweden	Retrospective cohort	38 (of 43)	Lip	Early	SCC (subset of mixed)	Serious	BT-PDR	Toxicity (clinical, non-standardised); cosmesis (non-validated)

SCC—squamous cell carcinoma; NR—not reported. ROBINS-I: overall risk-of-bias rating. Lang 2021 [28] was published online in 2021 (print issue *Tumori* **2022**, *108*, 125–133 [28]); the study label follows the online publication year. RT—radiotherapy; BT—brachytherapy (HDR/LDR/PDR—high/low/pulsed dose rate); EBRT—external beam radiotherapy; QoL—quality of life.

**Table 2:** Reported radiotherapy practices, doses and fractionation.

Study	Modality	Intent	Technique	Total Dose	Fractionation	Source	Elective Nodal Irradiation
Lang 2021 [28]	EBRT	Adjuvant	IMRT 15/19; 3D-CRT 4/19	66 Gy (primary); 54 Gy (nodes)	Conventional	Photons	Yes (94.7%)
Mut 2016 [29]	BT-HDR	Definitive	Interstitial	45 Gy	9 fx, BID (5 Gy/fx)	Ir-192	No (N0)
Guibert 2011 [24]	BT-LDR	Definitive postop.	Interstitial	65 Gy (mean; 55–77)	Continuous	Ir-192	No (‘wait-and-see’)
Lock 2011 [30]	BT-LDR	Definitive	Permanent implant	55 Gy	—	Au-198 grains	No
de Visscher 1999 [25]	NR (RT vs. surgery)	Definitive	NR	NR	NR	NR	NR
Pigneux 1979 [26]	BT-LDR	Definitive	Interstitial	NR	NR	Historical (Ra/Ir)	NR
Stoddart 1964 [27]	EBRT + BT-LDR	Definitive	Orthovoltage + interstitial	NR	NR	Orthovoltage/Ra	NR
El Ayachy 2021 [22]	BT-PDR	Definitive postop.	Interstitial	70.14 Gy (median; 60–85)	0.83 Gy/pulse, q2h	Ir-192	NR (multimodal rec.)
Johansson 2011 [23]	BT-PDR	Definitive postop.	Interstitial	60 Gy (median; 55–66)	0.83 Gy/pulse, q2h	Ir-192	No (N0)

EBRT—external beam radiotherapy; BT—brachytherapy; LDR/HDR/PDR—low/high/pulsed dose rate; BID—twice daily; fx—fractions; NR—not reported.

**Table 3:** Oncological outcomes, toxicity and functional/aesthetic outcomes.

Study	Local Control	DFS/RFS	Overall Survival	Median FU (mo.)	Acute Toxicity	Late Toxicity	Functional/ Aesthetic
Lang 2021 [28]	100% (LDFS 5 y)	85.7% (PFS 5 y)	61.5% (5 y)	62.4	G1–2 mucositis/xerostomia (G3 minority)	No G3/4	Functional (non-validated)
Mut 2016 [29]	96.9% (5 y)	85.7% (5 y)	61.4% (5 y)	56.4	Mucositis G ≥ 3 (all)	No G3/4	Cosmetic 94% good
Guibert 2011 [24]	89.1% (crude)	83% (5 y)	NR	64.8	No G3/4 (16.3% any)	No G3/4	Functional 99%; cosmetic 92%
Lock 2011 [30]	97.9% (2 y)	NR	NR	27	NR	No G3+	Cosmesis ‘good’
de Visscher 1999 [25]	‘Comparable’ (NR)	NR	NR	NR	NR	NR	NR
Pigneux 1979 [26]	95.5% (timepoint NR)	NR	NR	NR	NR	NR	NR
Stoddart 1964 [27]	85.3% (crude)	NR	NR	NR	NR	NR	NR
El Ayachy 2021 [22]	92.8% (2–4 y)	69.4% (PFS 4 y)	66.4% (4 y)	35.4	Radiomucositis G ≥ 2 (all)	Fibrosis G2 8.3%	FACT-H&N 127/148; TOI 84
Johansson 2011 [23]	94.5% (5–10 y)	86.4% (5–10 y)	58.9% (5 y)	55	Mucositis (clinical, all)	Necrosis 2%/ORN 2%	Cosmetic very good

LDFS—local disease-free survival; PFS—progression-free survival; FU—follow-up; ORN—osteoradionecrosis; NR—not reported. Timepoints and calculation methods (crude vs. actuarial) differ across studies.

**Table 4:** Risk-of-bias assessment (ROBINS-I), by domain.

Study	D1	D2	D3	D4	D5	D6	D7	Overall
S01 Lang 2021 [28]	Serious	Mod.	Low	Low	Mod.	Mod.	Low	Serious
S04 Mut 2016 [29]	Serious	Mod.	Low	Low	Mod.	Mod.	Low	Serious
S08 Guibert 2011 [24]	Serious	Mod.	Low	Low	Mod.	Low	Low	Serious
S09 Lock 2011 [30]	Mod.	Mod.	Low	Low	Low	Mod.	Low	Mod.
S13 de Visscher 1999 [25]	Serious	Serious	Serious	NI	Serious	Serious	Serious	Serious
S14 Pigneux 1979 [26]	Serious	Serious	Serious	NI	Serious	Serious	Serious	Serious
S15 Stoddart 1964 [27]	Serious	Serious	Serious	NI	Serious	Serious	Serious	Serious
S16 El Ayachy 2021 [22]	Serious	Mod.	Low	Low	Mod.	Mod.	Low	Serious
S17 Johansson 2011 [23]	Serious	Mod.	Low	Low	Low	Mod.	Mod.	Serious

D1 confounding; D2 selection of participants; D3 classification of interventions; D4 deviations from intended interventions; D5 missing data; D6 measurement of outcomes; D7 selective reporting. Low (green); Mod. = moderate (yellow); Serious (red); NI = no information (grey).

**Table 5:** Concordance of the principal findings of the review with the recommendations of the major international guidelines.

Finding of the Review	Guideline Recommendation	Concordance
Surgery and radiotherapy/brachytherapy offer comparable local control in early stages	NCCN: surgery preferred, definitive RT an alternative [7]. ESMO: modalities equivalent in small tumours [8]	Concordant
Interstitial brachytherapy—a curative, organ-preserving option in lip tumours	GEC-ESTRO/ACROP: brachytherapy recognised as a curative option in head and neck SCC and cutaneous cancers [18,31,32]	Concordant
Nodal ‘wait and see’ in T1–T2 N0 disease, with elective irradiation only in N+	NCCN: no routine elective neck treatment in mucosal lip T1–T3 N0 [7]; ESMO: decision guided by depth of invasion [8]	Concordant; selection criteria still non-uniform
Adjuvant radiotherapy in advanced cases with positive margins (Lang 2021 [28])	NCCN/ESMO: postoperative RT 60–66 Gy for risk features; chemo-RT with cisplatin for positive margins/extranodal extension [7,8]	Concordant
Unclarified role of concurrent chemotherapy in lip SCC	Indication for concurrent cisplatin limited to positive margins/extranodal extension [7,8]	Site-specific evidence gap

## Data Availability

All data supporting the conclusions of this review derive from the included published studies, cited in the reference list. The data-extraction forms and the complete list of excluded studies (with reasons for exclusion) are available as Supplementary Materials.

## Supplementary Materials

The following supporting information can be downloaded at: https://www.mdpi.com/article/10.3390/jcm15187090/s1, Supplementary Document S1: PICO(S) Framework; Supplementary Document S2: PROSPERO Registration; Supplementary Document S3: Search Strategy (verbatim search strings for PubMed/MEDLINE, Scopus, Cochrane Library Trials Register and ClinicalTrials.gov); Supplementary Document S4: PRISMA 2020 Framework (flow-diagram node data, Figure S1: PRISMA 2020 flow diagram, and the 27-item PRISMA 2020 reporting checklist); Supplementary Document S5: Data Extraction (methodology and data items); Supplementary Document S6: Screening Strategy and Excluded Studies (E01–E18); Supplementary Document S7: Additional Figures, Figure S2: ROBINS-I distribution of the risk of bias across the seven domains and the overall assessment, Figure S3: evidence map of the included studies by radiotherapy modality, decade and cohort size. Accompanying data files provided separately: Data-Extraction Workbook (REVIEW_BUZA_Data_Extraction_v5.xlsx); Excluded Studies Workbook (REVIEW_BUZA_Excluded_Studies.xlsx); PROSPERO registration record as downloaded from the platform (REVIEW_BUZA_PROSPERO_record.pdf, Version 1.1, 9 July 2026).

## Abbreviations

The following abbreviations are used in this manuscript:

SCC	squamous cell carcinoma
EBRT	external beam radiotherapy
BT	brachytherapy
LDR/HDR/PDR	low/high/pulsed dose rate
IMRT	intensity-modulated radiotherapy
VMAT	volumetric-modulated arc therapy
3D-CRT	three-dimensional conformal radiotherapy
LC	local control
DFS	disease-free survival
RFS	recurrence-free survival
PFS	progression-free survival
OS	overall survival
ORN	osteoradionecrosis
CTV	clinical target volume
ENI	elective nodal irradiation
ECE	extranodal (extracapsular) extension
ROBINS-I	Risk Of Bias In Non-randomised Studies of Interventions
SWiM	Synthesis Without Meta-analysis
PRISMA	Preferred Reporting Items for Systematic Reviews and Meta-Analyses
FACT-H&N	Functional Assessment of Cancer Therapy—Head and Neck
EQD2	equivalent dose in 2-Gy fractions
NCCN	National Comprehensive Cancer Network
GEC-ESTRO	Groupe Européen de Curiethérapie—European Society for Radiotherapy and Oncology
