# Supplementary material for: Radiotherapy in Squamous Cell Carcinoma of the Lip: A Systematic Review of Reported Practices, Clinical Outcomes, and Gaps in Consensus
Source: J Clin Med. 2026 Sep 12;15(18):7090. doi: 10.3390/jcm15187090 (PMC13607407; doi:10.3390/jcm15187090)
Supplement: Supplementary file 1 [file jcm-15-07090-s001.zip › REVIEW_BUZA_Supplementary_Materials/REVIEW_BUZA_PROSPERO_record.pdf]

NIHR | National Institute for Health and Care Research

PROSPERO
International prospective register of systematic reviews

Radiotherapy in Squamous Cell Carcinoma of the Lip: A Systematic Review of Reported Practices, Clinical Outcomes, and Gaps in Consensus

Oana Ciobanescu, Razvan Marian Balan

REVIEW TITLE AND BASIC DETAILS

Review title

Radiotherapy in Squamous Cell Carcinoma of the Lip: A Systematic Review of Reported Practices, Clinical Outcomes, and Gaps in Consensus

Condition or domain being studied

Lip neoplasm; Squamous Cell Carcinoma Of Skin; Head And Neck Cancer; Radiation Oncology And/or Radiotherapy

Rationale for the review

Squamous cell carcinoma of the lip occupies an ambiguous position in oncological classification, being variably managed within cutaneous or oral cavity radiotherapy frameworks depending on tumor location, clinical phenotype, and the subspecialty involved. This ambiguity results in significant heterogeneity in radiotherapy practice, including indications for adjuvant treatment, target volume delineation, dose prescription, and nodal management. Unlike other head and neck subsites, lip cancer lacks dedicated radiotherapy guidelines, and no systematic review specifically addressing radiotherapy practices and outcomes in this anatomical subsite has been identified. The present review aims to systematically characterize the available evidence, document the extent of practice variability, and identify the gaps in consensus that currently prevent standardization of radiotherapy for lip squamous cell carcinoma.

Review objectives

To identify and synthesize the available evidence on radiotherapy — adjuvant, definitive, and brachytherapy-based — in squamous cell carcinoma of the lip.

To characterize the variability in reported indications for adjuvant radiotherapy, including histopathological risk factors that justify treatment.

To document the variability in target volume delineation and elective nodal irradiation practices across published studies.

To summarize reported dose and fractionation schedules used in this anatomical subsite.

To synthesize available data on clinical outcomes including local control, disease-free survival, and overall survival.

To describe reported acute and late radiotherapy-induced toxicity, with attention to cutaneous and mucosal toxicity at the lip and vermilion.

To identify gaps in current evidence and propose directions for future prospective research.

Keywords
Lip cancer; Lip squamous cell carcinoma; Radiotherapy; Brachytherapy; Adjuvant Radiotherapy; Head and Neck Cancer; Target volume delineation; radiation toxicity; mucocutaneous toxicity; Multimodal treatment; practice variability; Clinical outcomes

Country
Romania

ELIGIBILITY CRITERIA

Population

Included
Adult patients aged 18 years or older with histologically confirmed squamous cell carcinoma of the lip, including lower lip, upper lip, and oral commissure, of any TNM stage, treated with radiotherapy.

Excluded
Patients with non-squamous cell carcinoma histology (basal cell carcinoma, melanoma, Merkel cell carcinoma) unless data for the squamous cell carcinoma subset are separately extractable. Patients under 18 years of age.

Intervention(s) or exposure(s)

Included
Radiotherapy; Brachytherapy; Chemoradiotherapy

External beam radiotherapy (any technique, including 2D conventional, 3D conformal, IMRT, VMAT, or IGRT) or brachytherapy, administered as adjuvant treatment following surgical resection or as definitive (primary) treatment in patients not undergoing surgery.

Excluded
Studies reporting exclusively dosimetric outcomes without any clinical outcome data. Studies in which radiotherapy was used solely for palliation without reported outcomes.

Comparator(s) or control(s)

Included
PICO tags selected: Surgery; Usual Care

No mandatory comparator is required for inclusion. Studies with a single treatment arm are eligible. Where comparators are reported — such as radiotherapy versus surgery alone, radiotherapy versus no adjuvant treatment, or comparisons between radiotherapy techniques — comparative data will be extracted and analyzed separately.

Study design

Both randomized and nonrandomized study types will be included.

Included
Randomised controlled trials, prospective cohort studies, retrospective cohort studies, and case series with a minimum of 10 patients reporting clinical outcomes of radiotherapy in squamous cell carcinoma of the lip.

Excluded
Case reports with fewer than 10 patients, review articles, editorials, letters, conference abstracts without full data, and purely dosimetric studies without clinical outcome data.

Context
Studies conducted in any clinical setting, including tertiary oncology centers, radiation oncology departments, and multidisciplinary head and neck cancer units. No geographic restrictions will be applied. Studies from any country and any time period will be considered eligible. Both academic and community hospital settings are included. No restrictions are applied based on healthcare system type or resource level.

TIMELINE OF THE REVIEW

Date of first submission to PROSPERO
24 March 2026

Review timeline
Start date: 20 March 2026. End date: 20 December 2026.

Date of registration in PROSPERO
24 March 2026

AVAILABILITY OF FULL PROTOCOL

Availability of full protocol
A full protocol has been written but is not available because:

The full protocol has been written as an internal working document but has not yet been submitted for publication. It will be made available upon reasonable request to the corresponding author.

SEARCHING AND SCREENING

Search for unpublished studies
Only published studies will be sought.

Main sources that will be searched
The main databases to be searched are *MEDLINE*, *PubMed* and *Scopus*.

Other important or specialist databases that will be searched
Cochrane Library Trials Register; ClinicalTrials.gov. Embase was not accessible to the review team and represents a recognised limitation.

Search language restrictions

The review will only include studies published in English.

Search date restrictions

There are no search date restrictions.

Other methods of identifying studies

Other studies will be identified by: *reference list checking (backward citation searching)* and *searching trial or study registers*.

Link to search strategy

A full search strategy has been uploaded to PROSPERO. The PDF may be accessed through this link
https://www.crd.york.ac.uk/PROSPEROFILES/22a444e838a603d72266820596269869.pdf.

Selection process

Studies will be screened independently by at least two people (or person/machine combination) with a process to resolve differences.

Other relevant information about searching and screening

Searches were conducted across four databases: PubMed/MEDLINE (491 records), Scopus (895 records), the Cochrane Library Trials Register (196 records), and ClinicalTrials.gov (125 records), yielding 1,707 records before deduplication. Embase was not accessible to the review team; this represents a recognised limitation that will be reported transparently in the final manuscript. Title and abstract screening will be performed independently by two reviewers using Rayyan. Disagreements will be resolved by consensus or by a third reviewer. Full-text screening will follow the same process.

DATA COLLECTION PROCESS

Data extraction from published articles and reports

Data will be extracted independently by at least two people (or person/machine combination) with a process to resolve differences.

Authors will not be contacted for further information.

Study risk of bias or quality assessment

Risk of bias will be assessed using: *Cochrane RoB-2* and *ROBINS-I*

Data will be assessed independently by at least two people (or person/machine combination) with a process to resolve differences.

Additional information will **not** be sought from study investigators if required information is unclear or unavailable in the study publications/reports.

Reporting bias assessment

Risk of bias due to missing results will not be assessed

Certainty assessment

Certainty of findings will not be assessed

OUTCOMES TO BE ANALYSED

Main outcomes

1. Local control rate: defined as absence of local recurrence at the primary tumour site, reported at any time point available. 2. Disease-free survival: time from treatment completion to documented recurrence or death from any cause. 3. Overall survival: time from diagnosis or treatment to death from any cause. Effect measures will be reported as rates or survival estimates as provided in the included studies.

Additional outcomes

1. Acute and late radiotherapy-induced toxicity: graded using any validated scale (CTCAE, RTOG, or institutional), reported at any available time point, with attention to cutaneous and mucosal toxicity at the lip and vermilion. 2. Target volume delineation: clinical target volume characteristics and elective nodal irradiation practices as reported. 3. Dose and fractionation: total dose and fractionation schedules used. 4. Functional and aesthetic outcomes: any reported measure of lip function, oral competence, or aesthetic result following treatment.

PLANNED DATA SYNTHESIS

Strategy for data synthesis

Given the anticipated heterogeneity of included studies in terms of design, population characteristics, treatment techniques, and outcome reporting, data synthesis will be primarily narrative, organised thematically by outcome domain and following the SWiM reporting guideline. Quantitative meta-analysis will be performed only if three or more studies report the same outcome with sufficiently homogeneous and extractable data, using a random-effects model with I-squared statistic to quantify heterogeneity. If meta-analysis is not feasible, a structured narrative synthesis will be conducted.

CURRENT REVIEW STAGE

Stage of the review at this submission 1 change

Review stage	Started	Completed
Pilot work	✓	✓
Formal searching/study identification	✓	✓
Screening search results against inclusion criteria	✓	✓
Data extraction or receipt of IPD	✓	✓
Risk of bias/quality assessment	✓	✓
Data synthesis	✓	✓

Review status

The review is currently planned or ongoing.

Publication of review results

Results of the review will be published in English.

REVIEW AFFILIATION, FUNDING AND PEER REVIEW

Review team members

Dr Oana Ciobanescu (review guarantor and contact) ORCID: 0009-0008-3382-9604. UMF Craiova. Romania.

No conflict of interest declared.

Mr Razvan Marian Balan. UMF Craiova. Romania.

No conflict of interest declared.

Named contact

Dr Oana Ciobanescu (oana.rusu@umfcv.ro). ORCID: 0009-0008-3382-9604. UMF Craiova. Romania.

Review affiliation

University of Medicine and Pharmacy of Craiova; Emergency County Hospital of Craiova, Romania

Funding source

Review has no funding and no agreed support from an academic institution and is done in authors' own time.

Peer review

There has been no peer review of this planned review.

ADDITIONAL INFORMATION

Additional information 1 change

This review addresses a gap identified through preliminary systematic searches of PubMed and Scopus, which identified no existing systematic review specifically addressing radiotherapy in squamous cell carcinoma of the lip. The review is intended for submission to a peer-reviewed journal focused on head and neck radiation oncology. Embase access was not available to the review team at the time of registration; if access is obtained before data extraction begins, Embase will be searched and the PROSPERO record updated accordingly.

Amendment (see revision note): the review has been completed. Two minor deviations from the registered protocol occurred during conduct and are declared transparently in the manuscript Methods: (i) pulsed-dose-rate (PDR) brachytherapy was considered eligible post-registration, on the basis of its radiobiological equivalence to continuous low-dose-rate brachytherapy recognised by GEC-ESTRO; and (ii) concurrent systemic therapy was recorded contextually only and was not treated as a comparative axis, owing to insufficient data for meaningful synthesis. Risk of bias was pre-specified using RoB-2 for randomised trials and ROBINS-I for non-randomised studies; as no randomised controlled trials met eligibility, only ROBINS-I was applied. Certainty of the evidence was not formally assessed (e.g. by GRADE), given the narrative (SWiM) synthesis of heterogeneous, predominantly single-arm studies; this is reported as a limitation. The final searches (20 March 2026) identified 1,707 records; after deduplication and screening, nine studies were included.

Review conflict of interest

Declared individual interests are recorded under team member details.. No additional interests are recorded for this review.

Medical Subject Headings

Brachytherapy; Carcinoma, Squamous Cell; Classification; Consensus; Disease-Free Survival; Humans; Lip; Radiotherapy, Adjuvant; Radiotherapy, Intensity-Modulated; Risk Factors; Head and Neck Neoplasms; Lip Neoplasms

Revision note 1 change

Minor amendment: review completed. Added declaration of two minor protocol deviations (PDR eligibility; concurrent systemic therapy contextual only), clarification that only ROBINS-I was applied as no RCTs were identified, and a note that certainty of evidence was not formally assessed. Review stage updated. No changes were made to the pre-registered eligibility criteria, outcomes, or primary analytical approach.

SIMILAR REVIEWS

Check for similar records already in PROSPERO

None of the identified similar records address the topic of the present review. The three flagged registrations concern radiotherapy in hepatocellular carcinoma, endometrial cancer, and gynecologic pelvic malignancies respectively — all anatomically and oncologically unrelated to squamous cell carcinoma of the lip. No existing PROSPERO registration specifically addresses radiotherapy practices, target volume delineation, or clinical outcomes in lip squamous cell carcinoma.

PROSPERO version history 1 change

- Version 1.1, published 09 Jul 2026
- Version 1.0, published 24 Mar 2026

Disclaimer

The content of this record displays the information provided by the review team. PROSPERO does not peer review registration records or endorse their content.

PROSPERO accepts and posts the information provided in good faith; responsibility for record content rests with the review team. The guarantor for this record has affirmed that the information provided is truthful and that they understand that deliberate provision of inaccurate information may be construed as scientific misconduct.

PROSPERO does not accept any liability for the content provided in this record or for its use. Readers use the information provided in this record at their own risk.

Any enquiries about the record should be referred to the named review contact
