# Supplementary material for: Radiotherapy in Squamous Cell Carcinoma of the Lip: A Systematic Review of Reported Practices, Clinical Outcomes, and Gaps in Consensus
Source: J Clin Med. 2026 Sep 12;15(18):7090. doi: 10.3390/jcm15187090 (PMC13607407; doi:10.3390/jcm15187090)
Supplement: Supplementary file 1 [file jcm-15-07090-s001.zip › REVIEW_BUZA_Supplementary_Materials/REVIEW_BUZA_Supplementary_SUBMISSION.pdf]

SUPPLEMENTARY MATERIALS

Radiotherapy in Squamous Cell Carcinoma of the Lip: A Systematic Review of Reported Practices, Clinical Outcomes, and Gaps in Consensus

Oana Ciobănescu, Anda Elena Crişan, Răzvan Marian Bălan, Virgil Pătraşcu, Andrada-Nemesis Crişan, Cristiana-Iulia Dumitrescu, Mădălin-Cristian Moraru, Suzana Măceş, Mihai Popescu, Daniela Dumitrescu

PROSPERO: CRD420261349994 · Search date: 20 March 2026 · Reporting standard: PRISMA 2020

Contents

- Supplementary Document 1 — PICO(S) Framework
- Supplementary Document 2 — PROSPERO Registration
- Supplementary Document 3 — Search Strategy (verbatim search strings)
- Supplementary Document 4 — PRISMA 2020 Framework (flow diagram + 27-item checklist)
- Supplementary Document 5 — Data Extraction (methodology and data items)
- Supplementary Document 6 — Screening Strategy and Excluded Studies (E01–E18)
- Supplementary Document 7 — Additional Figures (risk-of-bias distribution; evidence map)

Accompanying data files (provided separately): Data Extraction Workbook (REVIEW_BUZA_Data_Extraction_v5.xlsx); Excluded Studies Workbook (REVIEW_BUZA_Excluded_Studies.xlsx); PROSPERO registration record as downloaded from the platform (REVIEW_BUZA_PROSPERO_record.pdf, Version 1.1, 09 July 2026).

Supplementary Document S1 — PICO(S) Framework

The following PICO(S) framework was pre-specified in the PROSPERO registration prior to data extraction and was used to define the eligibility criteria applied at all screening stages.

Element	Inclusion	Exclusion
Population (P)	Adults (≥18 years) with histologically confirmed squamous cell carcinoma (SCC) of the lip — lower lip, upper lip, or oral commissure; any TNM stage.	Patients <18 years; non-SCC histology (BCC, melanoma, Merkel cell) unless SCC-specific data separately extractable; lip not the primary site with non-extractable lip data.
Intervention (I)	Any radiotherapy modality: external beam (2D, 3D-CRT, IMRT, VMAT, IGRT, orthovoltage, electrons) or brachytherapy (LDR, HDR, PDR; any isotope), adjuvant or definitive.	Purely dosimetric studies without clinical outcomes; radiotherapy for palliation without reported clinical outcomes.
Comparator (C)	No mandatory comparator — single-arm studies eligible; comparative data extracted where reported.	Not applicable.
Outcomes (O)	Primary: local control, disease-free survival (DFS), overall survival (OS). Secondary: acute/late toxicity (validated scales), CTV, dose/fractionation, elective nodal irradiation, functional and aesthetic outcomes.	Studies not reporting at least one primary outcome.
Study design (S)	RCTs; prospective/retrospective cohorts; case series with ≥10 patients; English language; no date restriction.	Case reports (n<10); reviews, editorials, letters, commentaries; conference abstracts without full data; non-English publications.

Supplementary Document S2 — PROSPERO Registration

The review was prospectively registered with PROSPERO (International Prospective Register of Systematic Reviews, Centre for Reviews and Dissemination, University of York) before data extraction. Key registration fields are summarised below; the full record is publicly accessible at the URL provided.

The record was amended upon completion of the review (Version 1.1, published 09 July 2026). The amendment declares the two minor protocol deviations reported in the manuscript Methods (post-registration eligibility of pulsed-dose-rate brachytherapy; concurrent systemic therapy recorded contextually only), clarifies that — as no randomised controlled trials met eligibility — only ROBINS-I was applied, and notes that the certainty of the evidence was not formally assessed (for example, by GRADE), given the narrative synthesis of heterogeneous, predominantly single-arm studies; this is reported as a limitation. The complete registration record, as downloaded from the PROSPERO platform, is provided as a separate supplementary file (REVIEW_BUZA_PROSPERO_record.pdf).

Field	Details
Registration number	CRD420261349994
Date of registration	24 March 2026
Full record URL	https://www.crd.york.ac.uk/PROSPERO/view/CRD420261349994
Review guarantor	Dr Oana Ciobănescu · oana.ciobanescu@yahoo.com · ORCID 0009-0008-3382-9604
Co-investigator	Mr Răzvan Marian Bălan — UMF Craiova
Information sources	PubMed/MEDLINE; Scopus; Cochrane Library (Trials); ClinicalTrials.gov register. Embase not accessible (declared).
Risk-of-bias tool	ROBINS-I for non-randomised studies; Cochrane RoB 2 for RCTs if identified.
Data synthesis	Primarily narrative synthesis (SWiM); meta-analysis only if ≥3 sufficiently homogeneous studies (threshold not met).
Declared deviations	(1) PDR brachytherapy considered eligible post hoc (GEC-ESTRO radiobiological equivalence to LDR); (2) concurrent chemotherapy recorded contextually only, not as a comparative axis.
Funding / conflicts	No external funding; no conflicts of interest declared.

Supplementary Document S3 — Search Strategy

Systematic searches were conducted across four electronic sources from inception to 20 March 2026 by the first author (O.C.). No date restrictions were applied. Controlled vocabulary (MeSH) was combined with free-text terms in title/abstract fields. Embase was not accessible (no institutional subscription). The final search strings are reproduced verbatim below.

Source	Type	Date	Results (n)
PubMed/MEDLINE	Database	20 Mar 2026	491
Scopus	Database	20 Mar 2026	895
Cochrane Library (Trials Register)	Database	20 Mar 2026	196
ClinicalTrials.gov	Register	20 Mar 2026	125
Total (before deduplication)	—	—	1,707

S3.1 PubMed/MEDLINE (n = 491)

```
("Lip Neoplasms"[MeSH Terms] OR "lip cancer"[tiab] OR "lip carcinoma"[tiab] OR "lip
squamous cell carcinoma"[tiab] OR "carcinoma of the lip"[tiab] OR "lower lip cancer"[tiab]
OR "upper lip cancer"[tiab] OR "lip tumor"[tiab] OR "lip tumour"[tiab] OR
"vermilion"[tiab]) AND ("Radiotherapy"[MeSH Terms] OR "Brachytherapy"[MeSH Terms] OR
"Chemoradiotherapy"[MeSH Terms] OR "radiation therapy"[tiab] OR "radiotherapy"[tiab] OR
"brachytherapy"[tiab] OR "interstitial radiotherapy"[tiab] OR "interventional
radiotherapy"[tiab] OR "IMRT"[tiab] OR "VMAT"[tiab] OR "adjuvant radiation"[tiab] OR
"postoperative radiation"[tiab] OR "definitive radiation"[tiab])
```

S3.2 Scopus — TITLE-ABS-KEY (n = 895)

```
( TITLE-ABS-KEY ( "lip cancer" OR "lip carcinoma" OR "lip squamous cell carcinoma" OR
"carcinoma of the lip" OR "lower lip cancer" OR "upper lip cancer" OR "lip tumor" OR "lip
tumour" OR "lip neoplasm" ) ) AND ( TITLE-ABS-KEY ( "radiotherapy" OR "radiation therapy"
OR "brachytherapy" OR "interstitial radiotherapy" OR "interventional radiotherapy" OR
"IMRT" OR "VMAT" OR "adjuvant radiation" OR "postoperative radiation" OR "definitive
radiation" OR "chemoradiotherapy" ) )
```

S3.3 Cochrane Library (Trials Register) (n = 196)

```
(lip cancer OR lip carcinoma OR lip squamous cell carcinoma OR carcinoma of the lip OR
lower lip cancer OR upper lip cancer OR lip tumor OR lip tumour OR lip neoplasm OR
vermilion) AND (radiotherapy OR radiation therapy OR brachytherapy OR interstitial
radiotherapy OR IMRT OR VMAT OR adjuvant radiation OR postoperative radiation OR
definitive radiation OR chemoradiotherapy)
```

S3.4 ClinicalTrials.gov register (n = 125)

```
lip squamous cell carcinoma radiation
```

Note. ClinicalTrials.gov was searched to identify registered ongoing or completed trials not yet published; all 125 records were screened at title/description level, and no eligible unpublished completed trials with available results were identified.

Supplementary Document S4 — PRISMA 2020 Framework

This document provides the PRISMA 2020 flow-diagram node counts, the rendered flow diagram (Figure S1), and the 27-item PRISMA 2020 reporting checklist. The review used two identification streams: databases/registers and citation searching. Individually assessed full-text exclusions are documented by study (E01–E16) in Supplementary Document 6; the remaining full-text exclusions (predominantly non-English-language and full-text-not-retrieved records) and the citation-searching counts are reported in aggregate, as they were not tallied per category prospectively.

S4.1 Flow-diagram node data

Stage	n	Cumulative / note
Identification — PubMed/MEDLINE	491	491
Identification — Scopus	895	1,386
Identification — Cochrane Library (Trials)	196	1,582
Identification — ClinicalTrials.gov (register)	125	1,707
Total records identified	1,707	Databases 1,582 + register 125
Duplicate records removed (Rayyan)	196	1,511 unique
Records screened (title/abstract)	1,511	2 reviewers, Blind Mode
Records excluded at title/abstract	1,290	—
Reports sought for retrieval / assessed	221	—
Removed at full-text stage (not retrieved + excluded)	214	= 221 − 7 included from databases
· individually assessed & excluded, with reasons	16	E01–E16 (Suppl. Doc. 6)
· grouped exclusions (non-English; lip not separable)	aggregate	E17–E18
Studies included from databases/registers	7	S01, S04, S08, S09, S13, S14, S15
Studies included via citation searching	2	S16 El Ayachy 2021; S17 Johansson 2011
Studies included in the final review	9	7 + 2
Total participants across included studies	~3,904	dominated by S15 Stoddart 1964 (n=3,166)

S4.2 Figure S1 — PRISMA 2020 flow diagram

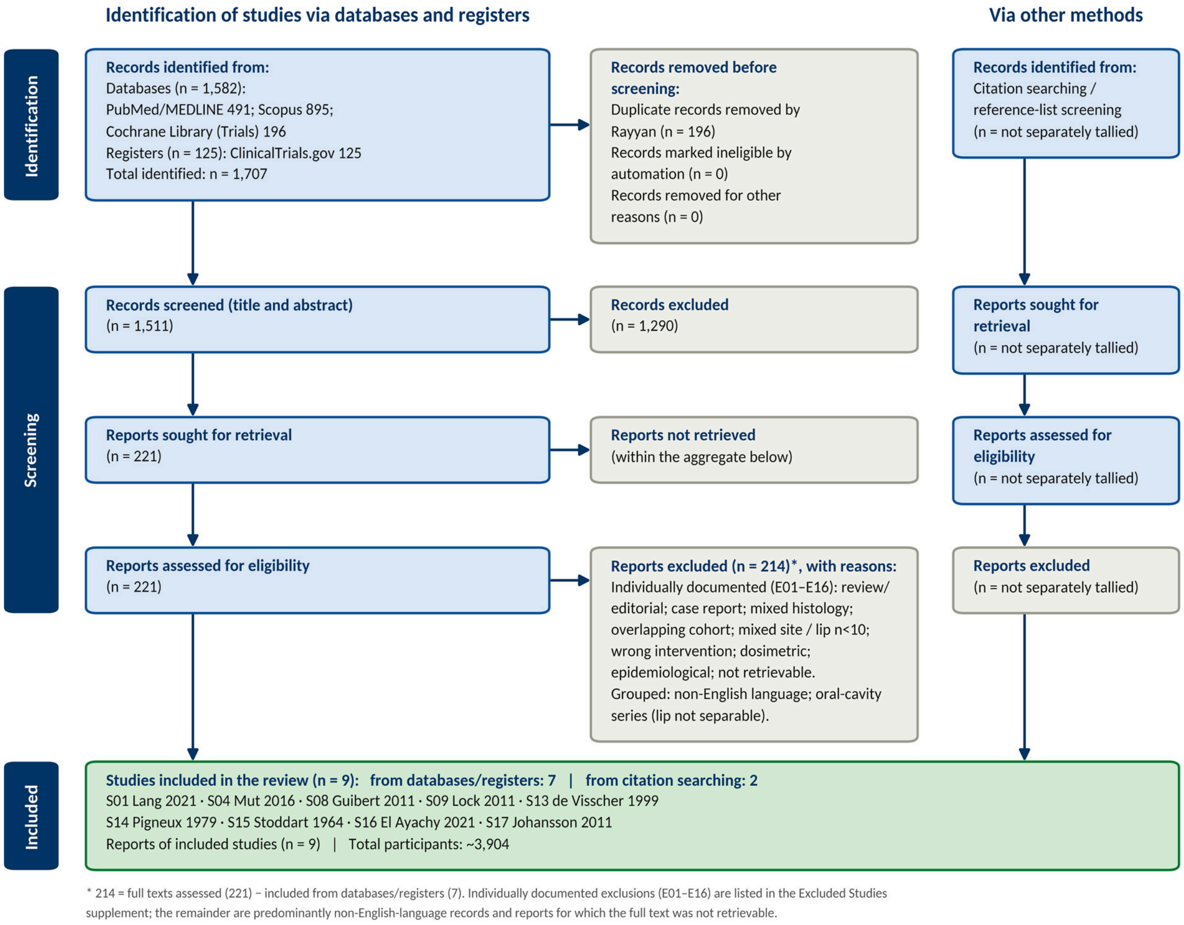

*Figure S1. PRISMA 2020 flow diagram (databases/registers and citation-searching streams). * 214 = full texts assessed (221) − included from databases/registers (7); individually documented exclusions E01–E16 are listed in Supplementary Document S6, the remainder reported in aggregate (E17–E18).*

S4.3 PRISMA 2020 reporting checklist (27 items)

#	Item	Where addressed
1	Title — identify as systematic review	Manuscript title
2	Abstract — structured summary	Manuscript abstract
3	Rationale	Introduction
4	Objectives (PICO)	PICO — Suppl. Doc. 1
5	Eligibility criteria	PICO (S1); Screening (S6)
6	Information sources	Search Strategy (S3): 3 databases + 1 register
7	Search strategy (full)	Search Strategy (S3) — all four sources
8	Selection process	Screening (S6): Rayyan, Blind Mode, 2 reviewers
9	Data collection process	Methods 2.5; Data Extraction Workbook
10	Data items (outcomes)	Data Extraction form (49 fields; S5)
11	Study risk of bias	ROBINS-I; Methods 2.6; Table 4
12	Effect measures	Narrative rates: LC%, DFS%, OS%
13	Synthesis methods	SWiM narrative synthesis; no meta-analysis
14	Reporting bias	Not formally assessed; discussed in limitations
15	Certainty assessment	No formal GRADE (pre-specified); rationale in limitations
16	Study selection	PRISMA flow (Figure S1)
17	Study characteristics	Table 1; Data Extraction Workbook

18	Risk of bias in studies	Table 4 (ROBINS-I)
19	Results of individual studies	Tables 2–3; main text
20	Results of syntheses	Results (per modality)
21	Reporting biases	Discussion — limitations
22	Certainty of evidence	Not formally assessed; narrative in Discussion
23	Interpretation	Discussion
24	Registration and protocol	PROSPERO CRD420261349994 (S2)
25	Support	No external funding
26	Competing interests	None declared
27	Availability of data	Supplementary Documents 1–7 + Excel workbooks

Supplementary Document S5 — Data Extraction

Data were extracted using a pre-specified form developed in Microsoft Excel (49 fields) and piloted on two studies before formal extraction. Extraction was performed by the first author (O.C.) and verified by the second author (R.M.B.). Where data appeared in both text and tables, table data were prioritised; where the abstract and the methods/results sections diverged, the methods/results sections were used. Fields not reported in the primary source were marked NR (not reported); no absent value was estimated or interpolated. The complete populated form is provided separately (REVIEW_BUZA_Data_Extraction_v5.xlsx), covering the nine included studies.

S5.1 Extracted data domains

Domain	Variables extracted
Study identification	Study ID, first author, year, journal, DOI/PMID, full-text access
Study design	Design, country, study period
Population	N total, N lip SCC, median age, sex, subsite, TNM distribution, histological confirmation
Prior treatment	Any prior surgery, radiotherapy, or systemic therapy
Intervention — radiotherapy	Modality, intent, technique, total dose, fractionation, dose per fraction, beam/source
Target volume	CTV description; elective nodal irradiation (Y/N + details)
Concurrent / timing	Concurrent systemic therapy; timing post-operative
Primary outcomes	Local control (%, timepoint, method); DFS/RFS; OS; regional control
Follow-up	Median and range (months)
Toxicity	Acute and late — scale used; G3+ description
Functional / aesthetic	Functional outcomes; aesthetic/cosmetic outcomes; validated QoL instruments
Risk of bias	ROBINS-I overall; domains flagged; notes/caveats

Supplementary Document S6 — Screening Strategy and Excluded Studies

All retrieved records (n = 1,707) were imported into Rayyan (new.rayyan.ai; Ouzzani M et al. Syst Rev 2016;5:210), with records from each source uploaded as separate files to preserve source traceability. Automated duplicate detection (title + author + DOI) removed 196 records, leaving 1,511 unique records. Both reviewers (O.C., R.M.B.) screened independently with Blind Mode enabled throughout; disagreements were resolved by consensus after unblinding, without a third reviewer.

S6.1 Eligibility criteria entered in Rayyan

Code	Domain	Criterion
IC 1	Population	Adults (≥18 y) with histologically confirmed SCC of the lip
IC 2	Subsite / stage	Any TNM stage — lower lip, upper lip, or oral commissure
IC 3	Intervention	Radiotherapy (adjuvant, definitive, or brachytherapy)
IC 4	Design	Original study: cohort, case series ≥10 patients, or RCT
IC 5	Outcomes	≥1 clinical outcome: local control, DFS, OS, or toxicity
EC 1	Wrong design	Case reports with fewer than 10 patients
EC 2	Wrong design	Reviews, editorials, letters, commentaries
EC 3	Wrong design	Conference abstracts without full published data
EC 4	Wrong histology	Non-SCC unless SCC data separately extractable
EC 5	Wrong intervention	Purely dosimetric studies without clinical outcomes
EC 6	Wrong population	Lip not primary site; lip data not separable
EC 7	Duplicate	Duplicate record identified at any stage

S6.2 Screening outcomes

Stage	n
Records imported (total)	1,707
Duplicate records removed (Rayyan)	196
Unique records after deduplication	1,511
Records excluded at title/abstract	1,290
Reports sought for retrieval / assessed for eligibility	221
Total removed at full-text stage (not retrieved + excluded)	214
· individually assessed & excluded, with reasons (E01–E16)	16
· grouped exclusions (E17–E18)	aggregate
Included from databases/registers	7
Included via citation searching (S16, S17)	2
Total studies included in the review	9

Note. The per-reason numeric breakdown of the 214 full-text exclusions (and the split between reports not retrieved and reports excluded after assessment) was not retained per category prospectively during screening; this is reported in aggregate per PRISMA 2020 guidance and acknowledged in the manuscript limitations.

S6.3 Individually documented exclusions (E01–E16) and grouped categories (E17–E18)

ID	Study	Reason for exclusion	Category
E01	Delannes / Rio 2013	Review article (PubMed 'Review'), not a primary study; originally S07; cited contextually in the Discussion.	Review / editorial
E02	Mercke 2024 (print: Head Neck 2025)	Mixed histology (78 SCC + 15 BCC + 8 other); SCC-specific outcomes not fully separable.	Mixed histology
E03	Ahmad 2017	Single case report (n = 1); technique description only; no quantitative contribution.	Case report (n<10)
E04	Torres-Quispe (NR)	Case report, n = 1, below the ≥10-patient threshold.	Case report (n<10)
E05	Guinot 2014	Overlapping cohort with S04/Mut 2016 (same institution, IVO Valencia); double-counting risk.	Overlapping cohort
E06	Unetsubo 2015	Mixed-site; only 2/17 patients (11.8%) had lip tumours; lip outcomes not separable (n<10).	Mixed site / lip n<10

E07	Ngan 2005	Mixed-site; only 4/13 patients (30.8%) had lip cancer; outcomes not reported by site.	Mixed site / lip n<10
E08	Vavassori 2012	Mixed oral cavity + lip; lip-specific n<10, not separable from the pooled cohort.	Mixed site / lip n<10
E09	Khalil 2008	Primary intervention is sentinel lymph-node biopsy, not radiotherapy; outside review scope.	Wrong intervention
E10	Feldman 2014	Primarily a dosimetric comparison; n = 7 (all T2); sample too small for efficacy estimates.	Dosimetric / insufficient n
E11	Ayerra 2010	Insufficient extractable data; mixed HDR+LDR cohort not separable by modality.	Insufficient data
E12	Farago 1963	Epidemiological study of oral-cavity cancer (betel-nut), not a radiotherapy outcomes study.	Wrong study type
E13	Masui 2020	Full text inaccessible despite repeated attempts; extraction impossible.	Full text not retrievable
E14	Bucur 2004	Full text inaccessible; no open-access version; extraction impossible.	Full text not retrievable
E15	Byars 1957	Not digitised/accessible (1957; no PMID/DOI); mixed-site; lip data unknowable.	Not retrievable / mixed site
E16	Del Regato 1948	'No abstract available' (PMID 18893283); full text inaccessible; no extractable data.	No abstract / no data
E17	Various (grouped)	Non-English-language publications, excluded per the English-only criterion (PICO-S); reported in aggregate.	Language (English only)
E18	Various (grouped)	Lip SCC within larger oral-cavity series; lip data not separable (n<10); reported in aggregate.	Population not separable

Supplementary Document S7 — Additional Figures

The following figures complement the main-text visualisations. They are exploratory and descriptive; they do not introduce new data beyond that reported in the included studies (main-text Tables 1–4).

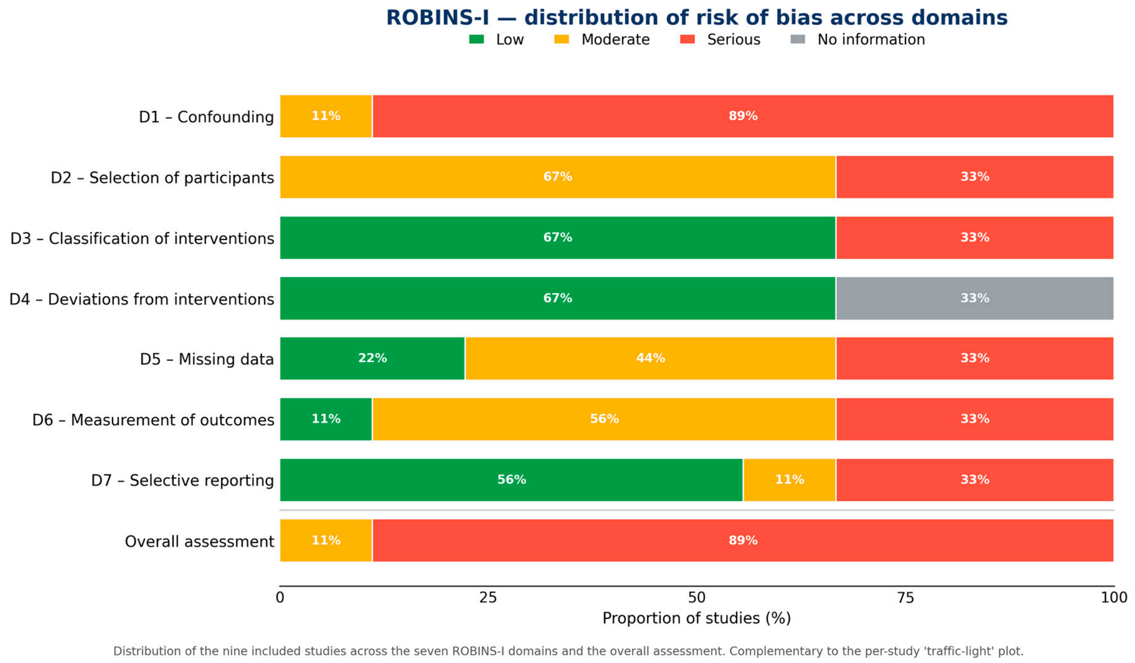

Figure S2. ROBINS-I — distribution of the risk of bias across the seven domains and the overall assessment, for the nine included studies (complementary to the per-study 'traffic-light' plot in main-text Figure 4).

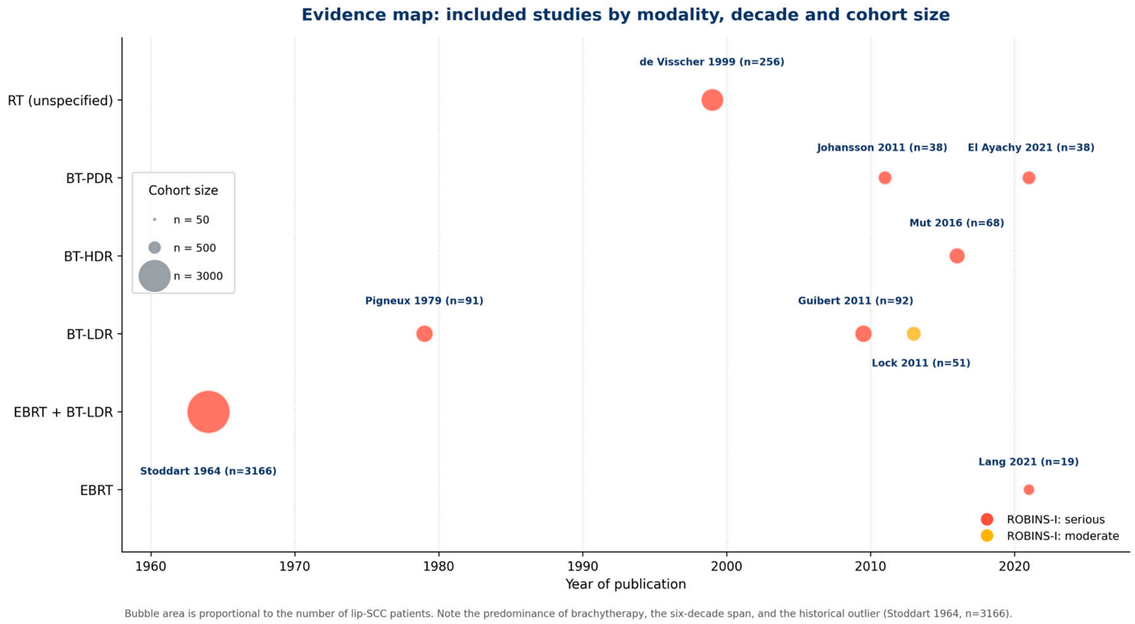

Figure S3. Evidence map of the included studies by radiotherapy modality, decade and cohort size. Bubble area is proportional to the number of lip-SCC patients; colour denotes the overall ROBINS-I rating.
